# Spatial halide exchange reactions for sculpting light in hybrid perovskites

**DOI:** 10.1039/d6ra06645c

**Published:** 2026-10-07

**Authors:** Md. Jahidul Islam, Ajoy Kumer

**Affiliations:** a Department of Chemistry, College of Arts and Sciences, IUBAT—International University of Business Agriculture and Technology Uttara Model Town Dhaka 1230 Bangladesh jahidul.chem@gmail.com; b Department of General Medicine, Saveetha Medical College and Hospitals, Saveetha Institute of Medical and Technical Sciences Chennai India ajoy.chem@iubat.edu

## Abstract

Hybrid perovskites have emerged as transformative materials in optoelectronics because their halide composition enables wide and controllable changes in bandgap and photoluminescence. Yet, translating this compositional tunability into spatially programmed optical functionality remains challenging. This review focuses specifically on spatially controlled halide exchange in hybrid and related lead–halide perovskites, emphasizing how the location, kinetics, and confinement of ion exchange determine the resulting optical landscape. We critically compare light-mediated/photocatalytic, laser-assisted, lithographic, solution–solid, solid–solid, and vapor-phase strategies in terms of localization mechanism, processing time, spatial resolution, compositional stability, scalability, and demonstrated functionality. Particular attention is given to vacancy-assisted ion migration, concentration gradients, interfacial diffusion, and optically driven precursor accumulation as the mechanistic basis of spatial control. We distinguish experimentally demonstrated outcomes—such as localized multicolor emission, compositionally graded nanowires, and single-crystal heterojunctions—from applications that remain prospective, including fully integrated micro-LED arrays and complex photonic circuits. The review identifies a central trade-off between spatial precision and throughput/stability and proposes standardized reporting of feature size, exchange time, compositional fidelity, and post-exchange stability as priorities for moving spatial halide exchange toward practical devices.

## Abstract 2

Hybrid perovskites have emerged as transformative materials in optoelectronics because their halide composition enables wide and controllable changes in bandgap and photoluminescence.

## Graphical abstract

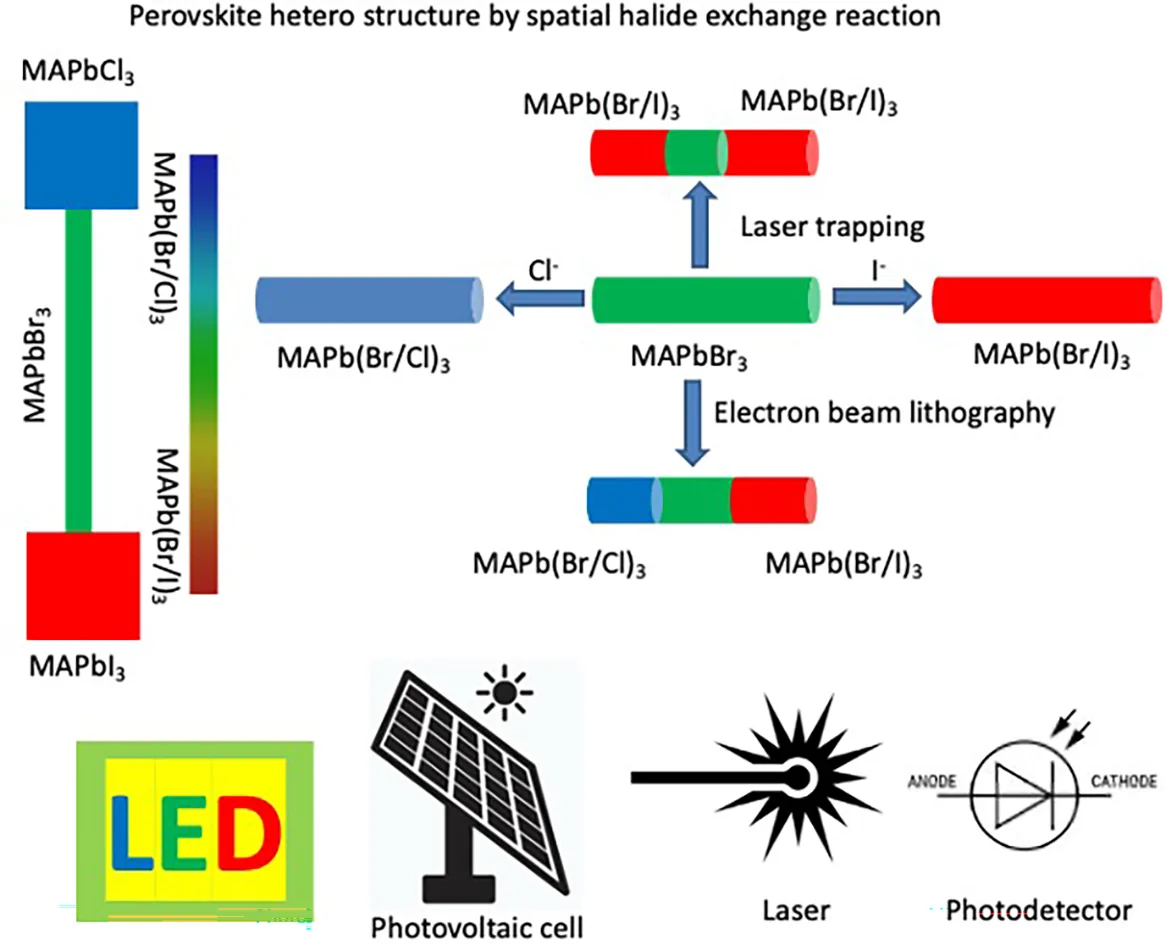

## 1. Introduction

Hybrid perovskites, characterized by the general chemical formula ABX_3_—where A represents an organic or inorganic cation, B is typically a divalent metal cation (such as Pb^2+^ or Sn^2+^), and X denotes a halide anion (Cl^−^, Br^−^, or I^−^)—have garnered intense research interest owing to their exceptional optoelectronic properties.^1,2^ The archetypal crystal structure of these materials, illustrated schematically in Fig. 1, comprises a three-dimensional framework of corner-sharing BX_6_ octahedra with A-site cations occupying the interstitial voids.^2^

**Fig. 1:**
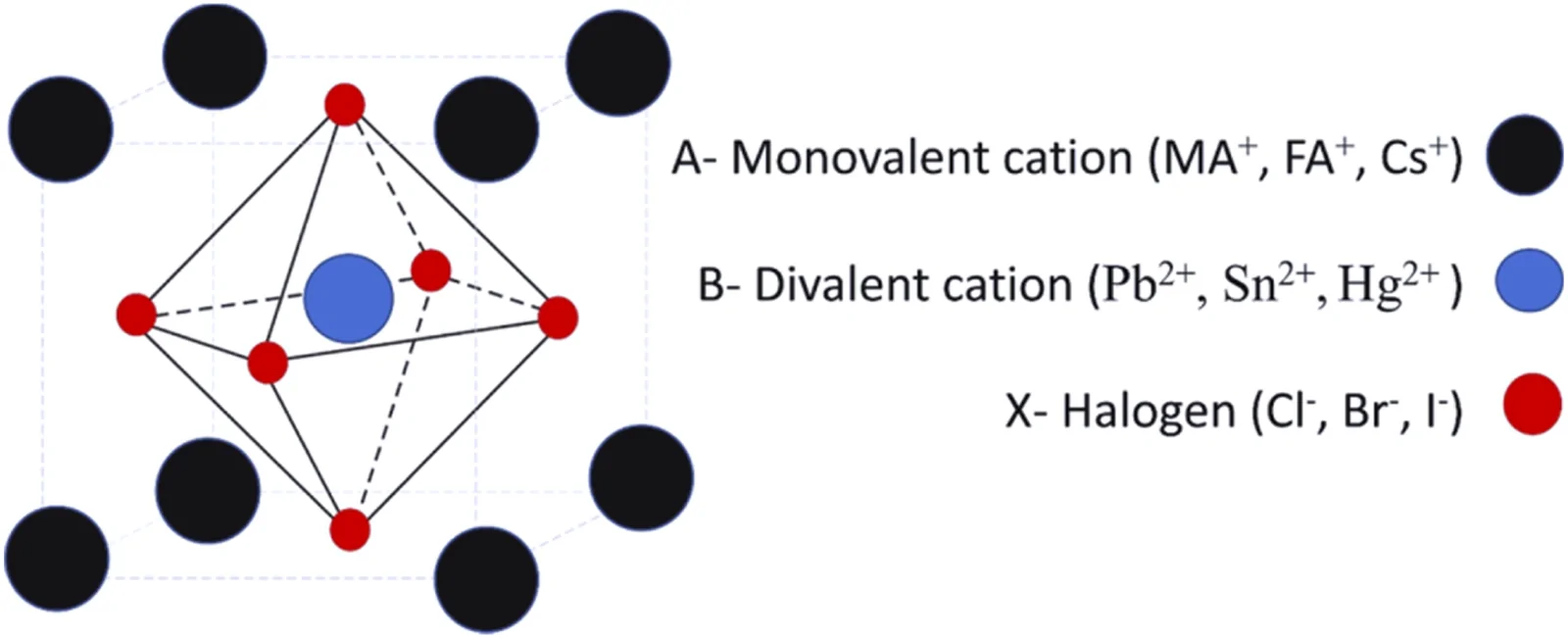
Visual representation of the ABX_3_ hybrid perovskite crystal structure.^2^ Reproduced from ref. 2 with permission from [Elsevier] [*Journal of Molecular structure*], copyright 2026.

These materials exhibit strong and tunable optical absorption spanning the visible to near-infrared spectrum, adjustable bandgaps through compositional engineering, and impressive charge-carrier mobilities and diffusion lengths. Such properties position hybrid perovskites at the forefront of next-generation optoelectronic devices, including light-emitting diodes (LEDs), photovoltaic cells, photodetectors, and coherent light sources such as lasers.^2^

To achieve optimal device performance, precise control over the perovskite's bandgap is crucial, which can be influenced by bulk compositional variations.^3^ However, manipulating light emission at the microscale offers an avenue for further optimization and exploration.

Fig. 2 illustrates the impact of halide composition on the optical properties of a series of hybrid perovskite single crystals. Specifically, methylammonium lead bromide (MAPbBr_3_) is alloyed with varying concentrations of chloride (Cl^−^) and iodide (I^−^) ions to modulate its band structure.^4^ The transmittance spectra reveal that these compositional modifications induce noticeable shifts in the absorption edge, signifying alterations in the bandgap energy. As the iodide content increases, the absorption edge redshifts, corresponding to a narrower bandgap. Conversely, incorporating chloride induces a blueshift and results in a wider bandgap. These trends are attributed to the differing ionic radii and electro negativities of Cl^−^ and I^−^, which influence the perovskite lattice and the extent of orbital overlap between the halide and lead orbitals, thereby altering the electronic band structure.^5^

**Fig. 2:**
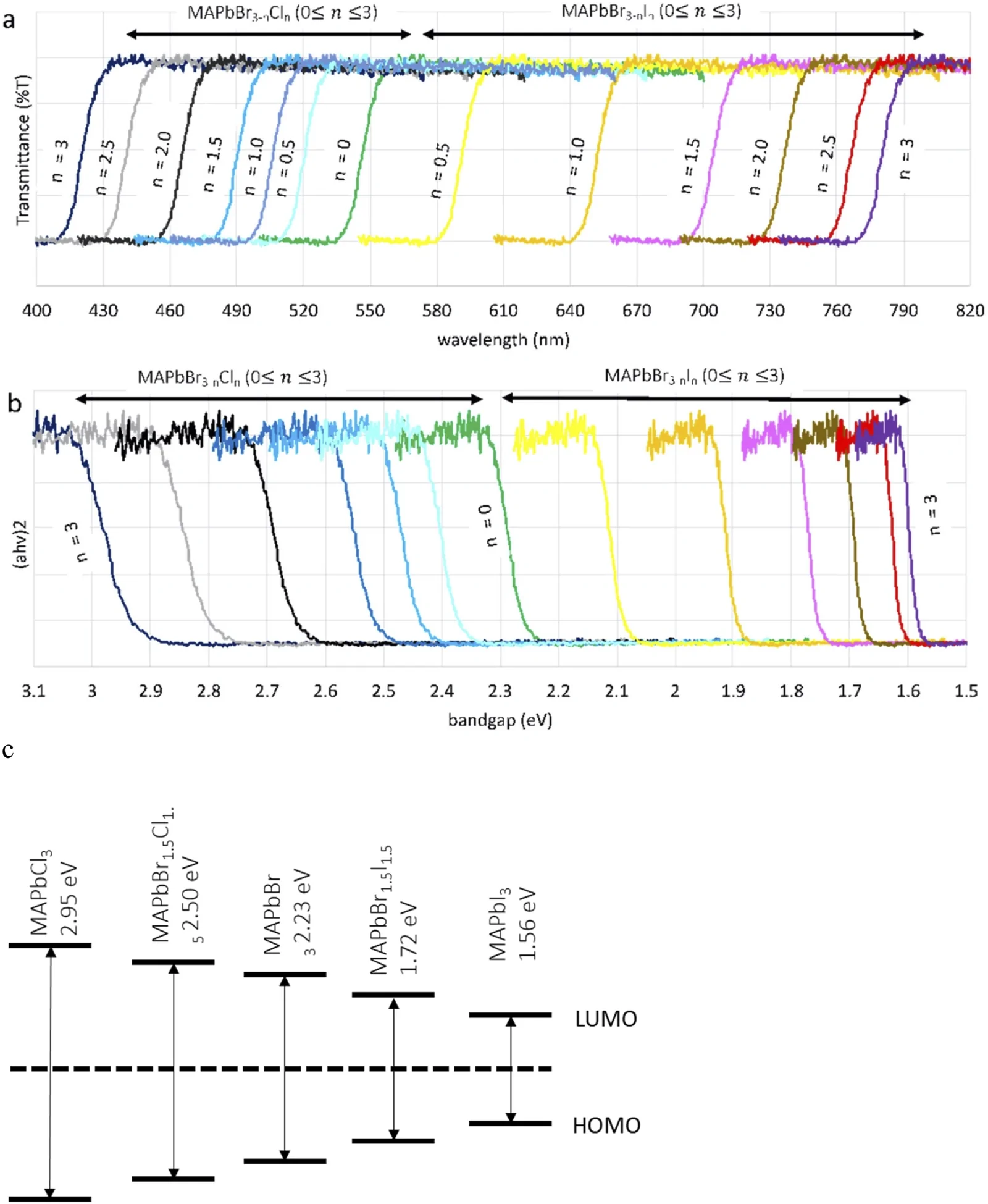
Optoelectronic properties tuning in hybrid perovskites *via* anion ratio change. (a) Transmittance spectra showing continuous band edge shifts for MAPbBr_3−*n*_Cl_*n*_ and MAPbBr_3−*n*_I_*n*_ systems (0 ≤ *n* ≤ 3). (b and c) Corresponding Tauc plots ((*αhν*)^2^*vs.* bandgap energy) showing systematic bandgap engineering from ∼1.6 eV to 3.0 eV, driven by halide composition change in the whole visible range.^6^ Reproduced from ref. 6 with permission from [Elsevier] [*Journal of Alloys and Compounds*], copyright 2026.

These bandgap shifts are further validated by energy values derived from Tauc plots based on the transmittance data. Such compositional control enables precise tuning of light absorption and emission characteristics, which is critical for tailoring perovskites for specific optoelectronic applications.^4^

Fig. 3 illustrates how the color of light emitted by perovskite single crystals changes depending on their chemical composition. The perovskite single crystals are represented as MAPbBr_3−*n*_X_*n*_ (X = Cl or I; 0 ≤ *n* ≤ 3).^6^ This variation in halide composition is a key method for tuning the optical properties of perovskites. All the crystals are excited with 405 nm UV light. The figure shows the distinct emission colors produced by these different perovskite compositions. By changing the ratio of halide ions (Br, Cl, and I) in the MAPbBr_3_ structure, the energy bandgap of the material is altered. This bandgap determines the wavelength (and thus the color) of the emitted light.^6^

**Fig. 3:**
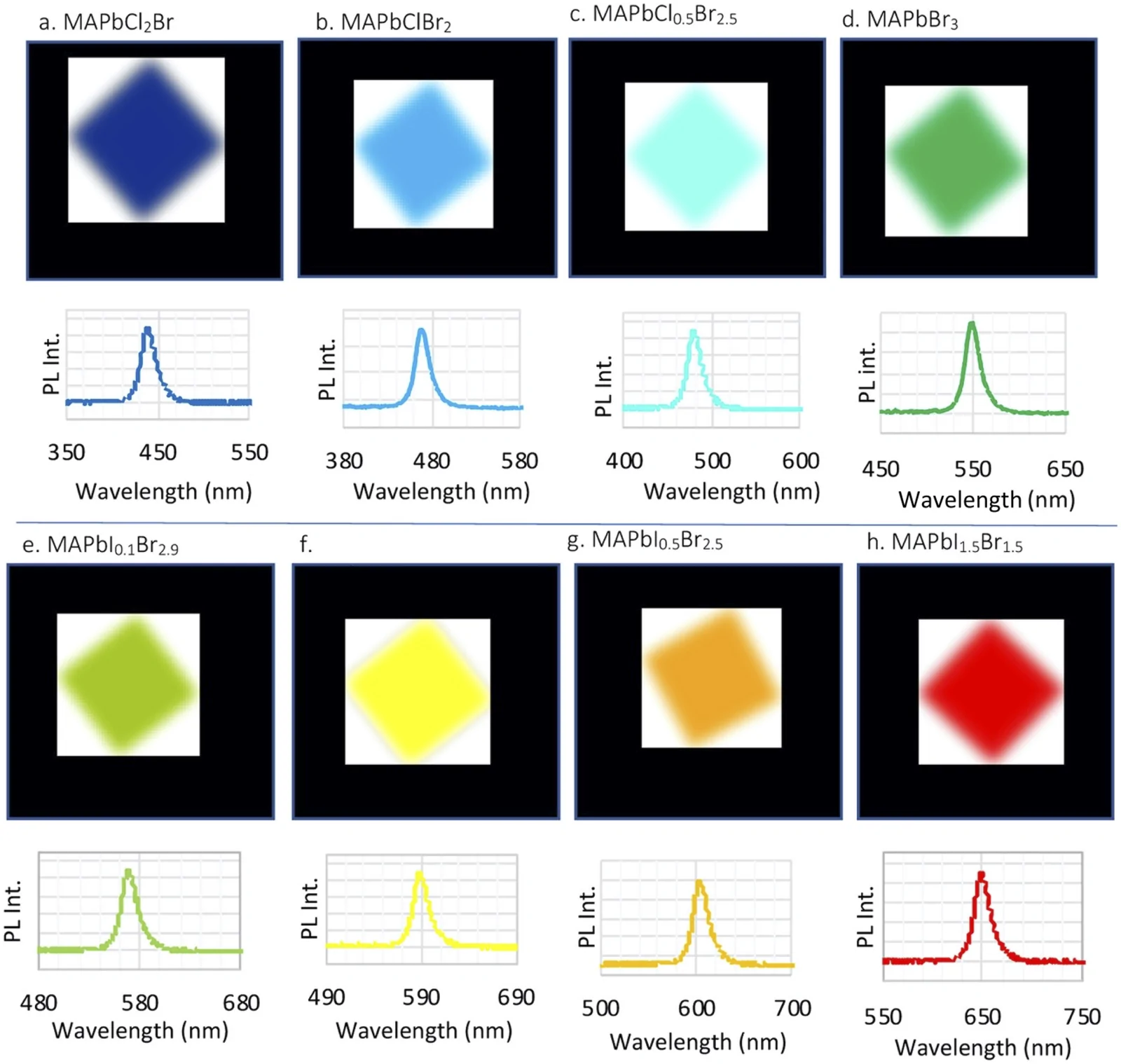
Emission color variation in perovskite single crystals due to compositional tuning. (a–h) show the distinct emission colors originating from different MAPbBr_3−*n*_X_*n*_ (X = Cl or I; 0 ≤ *n* ≤ 3) compositions when excited by 405 nm UV light with wide-field illumination.^6^ Reproduced from ref. 6 with permission from [Elsevier] [*Journal of Alloys and Compounds*], copyright 2026.

The precise tunability of emission color in perovskite materials is crucial for a range of advanced optoelectronic and biological applications.^7^ In LEDs and displays, this enables vibrant, accurate color reproduction with a wide color gamut, ideal for next-generation technologies. For lasers and photodetectors, high color purity allows precise wavelength selection and enhances sensitivity and selectivity.^8^ In solar cells, balancing color purity optimizes light harvesting efficiency while minimizing energy losses.^5^ In biological research, hybrid perovskites with tunable bandgaps and high color purity facilitate fluorescence imaging by producing clear, distinct images with reduced background noise, improving signal-to-noise ratios and image quality.^9^ Their customizable emission wavelengths also allow matching excitation to biological molecules, enhancing imaging sensitivity and specificity while reducing phototoxicity.^10,11^

Cesium Lead Bromide (CsPbBr_3_) perovskites, when engineered in various dimensionalities—such as 0D quantum dots, 1D nanowires, 2D nanosheets, and 3D bulk crystals—offer unique advantages for the bioimaging of HeLa cancer cells in aqueous environments, as their dimensionality directly affects optical properties, quantum confinement, and stability, enabling highly tailored photoluminescence for high-contrast, multiplexed cellular imaging.^12^ The electronic band structure of these lead halide perovskites is intricately influenced by the halide ion composition: the conduction band minimum arises mainly from the 6p orbitals of Pb^2+^, while the valence band maximum is a hybrid of Pb^2+^ 6s orbitals and halide (Cl^−^, Br^−^, I^−^) p orbitals, making the band structure highly sensitive to the type and proportion of halide ions present.^13^ By substituting different halides, the octahedral geometry of the perovskite lattice is altered due to ionic radii differences, which directly tunes the bandgap energy—enabling emission control across 1.6–3.1 eV, from visible to near-infrared.^14^ Bandgap modulation can be achieved through direct solution synthesis, where mixed halide precursors are crystallized with precise stoichiometry,^6^ or *via* post-synthetic halide exchange, where pre-formed perovskite undergo halide substitution through vacancy-assisted diffusion—a process characterized by low activation energy that allows efficient, reversible, and precise bandgap tuning under mild conditions without compromising crystal integrity.^14,15^

The Fig. 4 illustrates a simplified molecular orbital diagram showing the formation of valence and conduction bands in CsPbI_3_. The diagram starts with the atomic orbitals of the constituent elements: iodine(i) 5s and 5p orbitals, and lead (Pb) 6s and 6p orbitals. When these atoms combine to form the CsPbI_3_ perovskite structure, their atomic orbitals interact, splitting into bonding and antibonding orbitals.^13^ The bonding orbitals (π_pp_, σ_pp_) are lower in energy and contribute to the valence band, while antibonding orbitals 
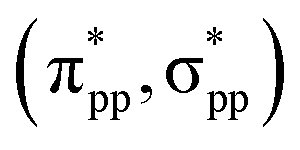
 are higher in energy and contribute to the conduction band. The π_pp_ → n_b_ represents a non-bonding orbital, which also contributes to the valence band. The valence band is formed from the higher energy bonding orbitals and the non-bonding orbital and is primarily composed of I 5p and Pb 6s orbitals.^16^ The conduction band is formed from the antibonding orbitals, mainly Pb 6p orbitals. The bandgap (*E*_g_) is the energy difference between the top of the valence band and the bottom of the conduction band. This energy difference represents the minimum energy required to excite an electron from the valence band to the conduction band, enabling electrical conductivity and light absorption. The figure directly shows the origin of the bandgap in CsPbI_3_. The energy levels of the atomic orbitals and their interactions determine the width of the valence and conduction bands, and consequently, the size of the bandgap. A smaller bandgap means that less energy is required to excite electrons, leading to absorption of lower energy light (longer wavelengths). A larger bandgap means that more energy is needed for excitation, resulting in absorption of higher energy light (shorter wavelengths). In CsPbI_3_, the specific arrangement and energies of the I and Pb orbitals, as illustrated in the diagram, lead to a particular bandgap energy.

**Fig. 4:**
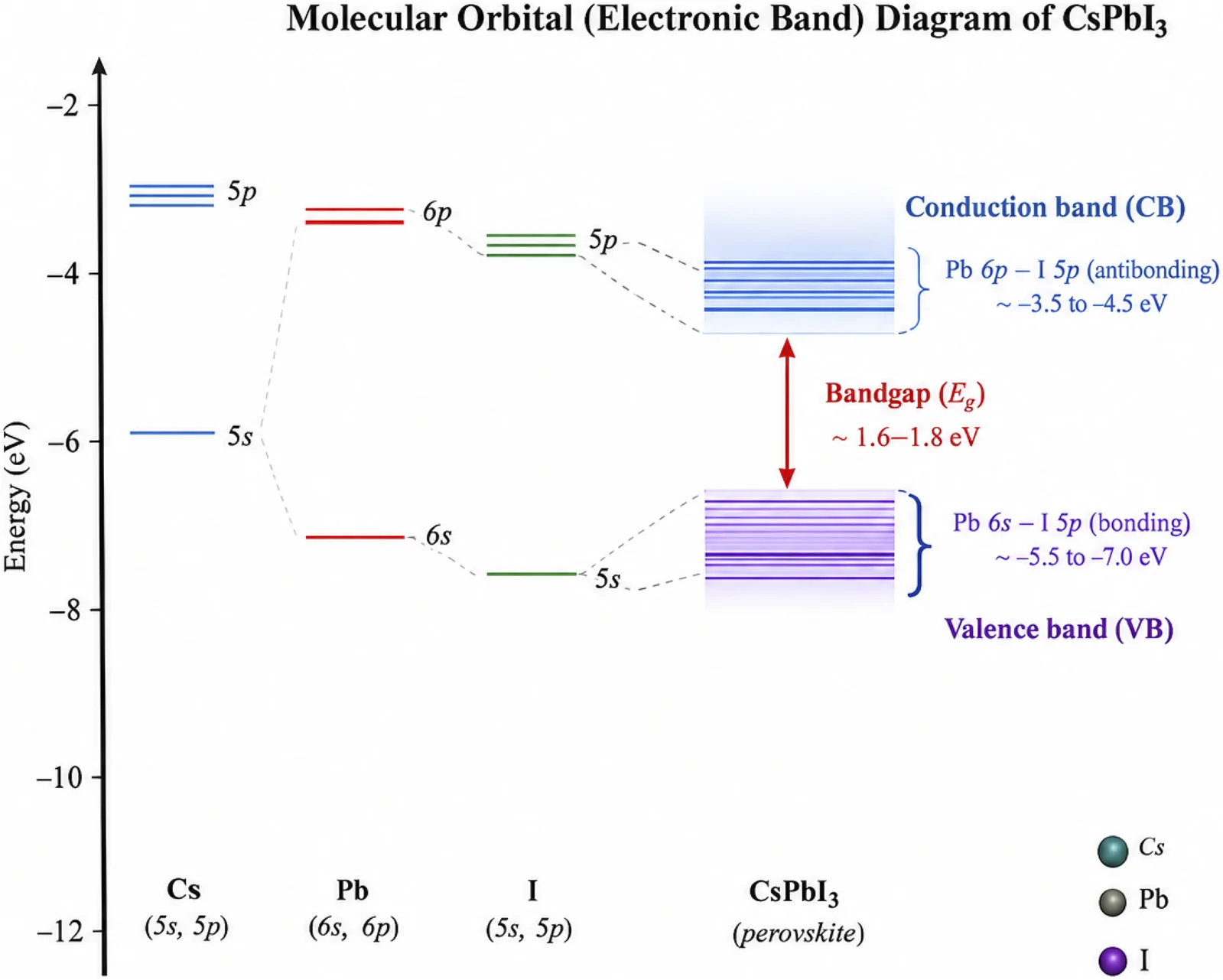
Molecular orbital mechanism of bandgap formation in CsPbI_3_. Simplified electronic schematic showing the orbital interactions between Pb and I atomic states. The hybridization of these frontier orbitals drives the formation of the valence band maximum (VBM) and conduction band minimum (CBM), establishing the fundamental optical bandgap (*E*_g_) of the perovskite semiconductor.

To illustrate how halide exchange evolves over time within a single perovskite crystal, the following figures combine both visual and spectral evidence. This approach highlights how emission color changes correlate with halide composition under controlled chemical exposure.

Fig. 5 demonstrates the time-dependent halide exchange process in a MAPbBr_3_ perovskite single crystal upon exposure to a methylammonium iodide (MAI) solution by qualitative and quantitative manner. Initially, at 0.1 minutes (a), the crystal exhibits a green emission characteristic of MAPbBr_3_. As the reaction progresses over 15 minutes (b), the emission shifts to yellow, indicating the partial substitution of bromide (Br^−^) ions with iodide (I^−^) ions, forming a mixed halide perovskite (MAPbBr_*x*_I_1−*x*_).^17^ Further exposure to MAI (c), also shown at 15 minutes but representing a later stage, leads to a red emission, signifying a higher concentration of iodide within the crystal lattice.^17^ This sequence of optical images directly illustrates the tunability of perovskite optical properties through halide exchange. Complementing this, the transmission microscopy (TM) image (d) reveals the well-defined, regular morphology of a pristine MAPbBr_3_ microcrystal, confirming its high crystalline quality before the halide exchange reaction commences.^18^

**Fig. 5:**
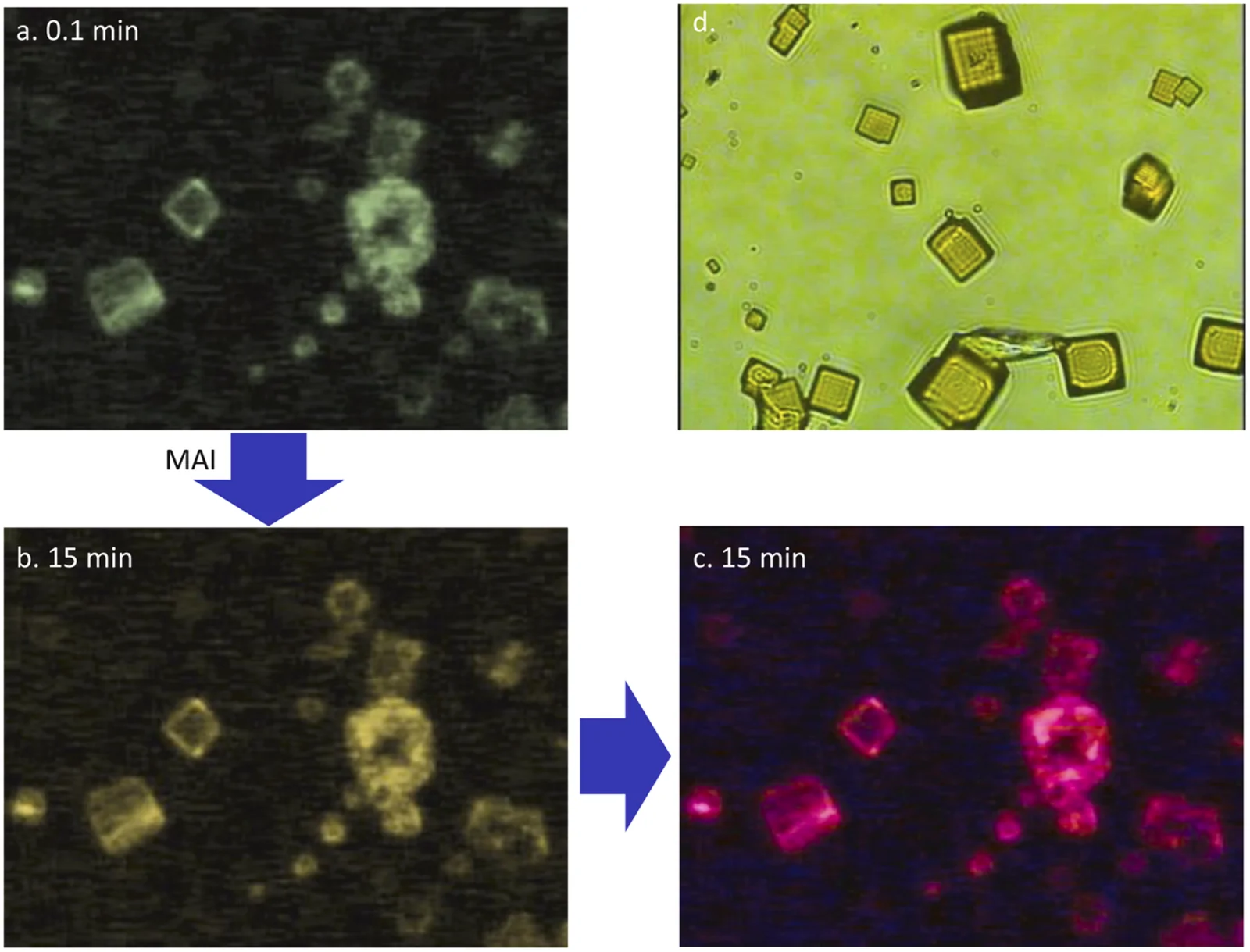
Dynamics of halide exchanges in MAPbBr_3_ perovskite single crystals. The (a–c) chronological optical microscopy series is tracking photoluminescence evolution during exposure to MAI solution. The emission shifts from green to yellow, and ultimately red (0.1 to 15 min), illustrating progressive bromide substitution by highly mobile iodide anions into the lattice vacancies. (d) Transmission microscopy (TM) characterization revealing the highly crystalline structural integrity of an unreacted, pristine MAPbBr_3_ microcrystal.^17^ Reproduced from ref. 17 with permission from [Elsevier] [*Chemical Physics Impact*], copyright 2026.

Controlled spatial halide exchange in hybrid perovskites is crucial for optimizing device performance and enabling site-specific functionalities, particularly in applications involving color patterning and wavelength-selective absorbance.^19,20^ While spontaneous (uncontrolled) halide exchange can naturally occur, it often leads to compositional drift, phase segregation, and ultimately performance degradation.^21^ Thus, precise control over halide composition is essential to achieve reproducible optoelectronic properties. Spatial halide exchange reactions offer transformative advantages by providing unprecedented control over halide distribution within a single perovskite crystal, enabling the creation of spatially distinct bandgaps and localized photoluminescence emission.^22,23^ This spatial tunability holds immense potential for revolutionizing optoelectronic applications.^22^

For example, in the development of next-generation micro-LED displays, traditional designs rely on arrays of individual subpixels (red, green, and blue) fabricated separately. Spatially patterned perovskites, on the other hand, allow different color emissions from a single-crystal substrate, potentially eliminating the need for color filters, enhancing energy efficiency, and enabling ultra-high-resolution displays.^24^

Table 1 highlights the contrast between spatial and uncontrolled halide exchange across several dimensions. Spatial halide exchange enables patterned light emission, micro- to nanoscale color tunability, and device-level customization—making it ideal for photonic circuits and micro-LEDs. However, it comes with trade-offs such as lower throughput, higher cost, and reliance on precision alignment and kinetic control.^24,25^ In contrast, uncontrolled halide exchange is simpler, more scalable, and suitable for bulk bandgap tuning in applications like solar cells, but it lacks spatial resolution and may result in phase separation and inconsistent performance.

**Table 1:** Comparison of spatial and uncontrolled halide exchange reaction in hybrid lead–halide perovskites

Aspect	Spatial halide exchange	Uncontrolled halide exchange
Definition	Controlled and localized replacement of halide ions in perovskite structures	Random and bulk-level halide ion substitution throughout the perovskite matrix
Advantages	- Enables patterned light emission – facilitates color tuning at micro/nanoscale – suitable for integrated photonics and micro-LEDs	- Simpler processing – useful for bulk tuning of optoelectronic properties
Disadvantages	- Requires sophisticated equipment – low throughput – higher cost	- Loss of spatial resolution – phase segregation and non-uniform properties
Technical difficulties	- Precise alignment of excitation source (*e.g.*, laser) – controlling reaction kinetics – requires clean, stable environment	- Inconsistent halide distribution – difficult to reverse or fine-tune post-reaction
Precautions during reaction	- Avoid high intensity that may degrade crystal – maintain inert atmosphere – careful control of ion source concentration	- Prevent overheating – monitor phase separation – minimize exposure to humidity
Scalability	- Currently limited to lab-scale – requires development of high-throughput techniques	- More scalable for bulk synthesis
Application suitability	- Micro-LEDs – photonic circuits – optical sensors	- Solar cells – bulk photodetectors
Stability of outcome	- More stable due to localized control	- Less stable due to ion migration and phase instability

Technically, spatial halide exchange requires controlled reaction atmospheres, laser alignment or photolithographic precision, and careful regulation of light intensity and temperature. Meanwhile, uncontrolled exchange primarily necessitates thermal stability, prevention of ion migration, and uniform precursor distributions.^26,27^ Although scalability currently favors uncontrolled methods, the ability of spatial halide exchange to provide precise, location-specific functionality makes it indispensable for advanced optoelectronic technologies.^28,29^

K. Sandeep, *et al.* summarized recent progress in ion exchange of halide perovskites, encompassing mechanisms, methods, and varying scales from nanoscale to bulk crystals. The review also discusses the potential of ion exchange for halide perovskites, aiming to benefit structural design and compositional optimization for diverse optoelectronic applications.^29^

Enzheng Shi *et al.* summarized recent progress in synthesizing halide perovskite epitaxial heterostructures, highlighting effective routes to stabilize these materials.^30^ Their work focuses on 2D halide perovskites, emphasizing the crucial role of organic ligands in enhancing thermal and environmental stability and suppressing halide interdiffusion. Molecular dynamics simulations and advanced microscopy were used to understand the stabilization mechanisms. Those strategies enable diverse heterostructures and superlattices with sharp interfaces due to reduced ion motion and improved stability.

While research on spatial or controlled halide exchange reactions in hybrid perovskites is still emerging, significant opportunities remain to advance this field.^31,32^ Further exploration in this area holds the potential to push the boundaries of optoelectronic technology by enabling the development of state-of-the-art devices based on these photo-functioning materials.

This review consolidates recent progress in spatially controlled halide exchange within hybrid perovskites, spanning nanocrystals, microcrystals, microrods, nanowires, and thin films. Its distinctive perspective is to treat spatial halide exchange not simply as a post-synthetic composition-tuning reaction, but as a coupled reaction–transport–patterning problem in which the localization of the halide source, the exchange phase, vacancy density, diffusion length, and reaction time determine the final optical map. Unlike broader reviews that primarily classify anion-exchange chemistry or perovskite device applications, we compare the spatial-control mechanisms themselves and evaluate what has actually been demonstrated at the microscale *versus* what remains a proposed device concept. We therefore use a unified framework based on material/morphology, exchange phase, halide source, localization method, spatial resolution, processing time, spectral response, stability evidence, and scalability. This framework is intended to clarify why highly localized laser-assisted exchange offers precision but limited throughput, whereas diffusion-, interface-, and vapor-based approaches can provide larger-area gradients at the cost of weaker confinement. The review also places spatial halide exchange in the broader context of halide-component and ion-migration strategies reported for perovskite optoelectronics, including recent work on lead-free and pseudohalide-containing perovskites.

## 2. Research methodology

The methodology adopted for this review systematically integrates literature published from 2010 to 2025. Primary research articles were identified through Web of Science, Scopus, ScienceDirect, and Google Scholar using combinations of the terms “hybrid perovskites,” “halide exchange,” “spatial control,” “laser trapping,” “photocatalytic exchange,” “lithography,” “microfluidics,” “solid-state diffusion,” “vapor-phase exchange,” and “optoelectronics.” Studies were included when they (i) demonstrated or directly analyzed halide exchange or halide redistribution in a perovskite material and (ii) provided a spatially resolved feature, gradient, localized reaction, patterned emission, heterojunction, or a clear mechanism relevant to spatial confinement. Reviews were used selectively for mechanistic and historical context. Studies were excluded from the core spatial-exchange comparison when they reported only homogeneous/bulk halide exchange, general bandgap tuning without spatial localization, or device improvements that could not be directly attributed to a spatially controlled exchange step. In particular, bulk solar-cell efficiency demonstrations and polymer–perovskite luminescent membranes without a halide-exchange localization step were not treated as evidence of spatial halide exchange.

The selected literature was thematically organized into four domains: exchange mechanisms, spatial-control strategies, demonstrated device functions, and scalability/stability. Each study was assessed for the identity and morphology of the perovskite, exchange phase, halide source, localization method, reported feature size or spatial resolution, processing time, spectral shift, stability evidence, and principal limitation. When a quantitative value was not reported in the source material, it is explicitly marked as “NR” (not reported) rather than inferred. This approach enables a critical comparison while avoiding the conflation of spatial halide exchange with conventional bulk anion exchange or with perovskite patterning methods that do not involve halide redistribution.

## 3. Fundamentals of halide exchange reactions in perovskites

Perovskite structures incorporate halide anions (Cl^−^, Br^−^, I^−^), which play a critical role in determining optical properties and structural stability.^33^ The size and electronegativity of the halide ions directly influence the bandgap, thereby dictating the material's light absorption and emission characteristics. For example, replacing iodide (I^−^) with smaller, more electronegative chloride (Cl^−^) results in a wider bandgap, shifting the absorption and photoluminescence spectra toward the ultraviolet region.^6^

Halide exchange reactions involve the diffusion and replacement of halide ions within the perovskite lattice. These dynamic processes allow fine-tuning of optical properties without altering the overall crystal structure.^6,23^ The efficiency and extent of halide exchange depend on multiple factors, including temperature, reaction time, and the choice of halide precursors.^34^ Generally, higher temperatures enhance halide ion mobility and facilitate more complete exchange, while precursor compatibility (*e.g.*, solubility and reactivity) ensures better incorporation and uniformity of halide ions.^35^

Understanding the role of halide anions (chlorine, bromine, iodine) in perovskites is crucial to appreciating the impact of halide exchange reactions. These anions occupy specific positions within the perovskite crystal lattice, influencing both the material's structure and properties. The size and electronegativity of the halide significantly affect the bandgap – the energy difference between occupied and unoccupied electronic states. Larger halides like iodine lead to a smaller bandgap, resulting in lower energy light absorption and red-shifted emission. Conversely, smaller halides like chlorine create a larger bandgap, favoring higher energy light absorption and blue-shifted emission.^36^ Additionally, the halide identity plays a role in perovskite stability. Chlorine-based perovskites generally exhibit higher stability, while those with iodine are more susceptible to degradation by moisture and light.^33^ Halide exchange reactions exploit these relationships, allowing researchers to tailor the bandgap and stability of perovskites by strategically replacing specific halides.

## 4. Mechanism of halide exchange: diffusion and replacement processes

Halide exchange reactions in perovskites involve the controlled substitution of halide anions (chlorine, bromine, iodine) within the crystal lattice. This manipulation alters the material's optoelectronic properties, offering a powerful tool for tailoring perovskite performance. The mechanism behind this exchange depends on two key processes: diffusion and replacement.^37^

Diffusion refers to the movement of halide ions within the perovskite crystal structure. This movement can be driven by a concentration gradient. When a perovskite crystal is exposed to a solution containing a different halide ion (*e.g.*, immersing a chlorine-based perovskite in a bromine solution), a concentration gradient is established. This gradient creates a driving force for the initial halide ions within the crystal (*e.g.*, chlorine) to diffuse outwards and be replaced by the more abundant halide ions (*e.g.*, bromine) from the surrounding solution.^22,38,39^

Replacement signifies the actual substitution of the original halide ion with the incoming one within the perovskite lattice. This replacement process often involves vacancy defects within the crystal structure. Vacancies are empty spaces where a halide ion would normally reside. The incoming halide ions can migrate towards these vacancies and occupy them, effectively replacing the original halide and altering the overall composition of the perovskite.^33^

The efficiency of ion diffusion and replacement in halide perovskites depends on factors such as temperature, ion size and charge, and crystal defects. Defects play a crucial role by providing pathways that lower the activation energy for ion migration, enhancing conductivity and material performance.^40^ Solution-processed perovskites often have high defect densities due to rapid crystallization, affecting optoelectronic properties, while single crystals exhibit fewer defects and better charge transport. In materials like MAPbI_3_, vacancies of iodide (I^−^), lead (Pb^2+^), and methylammonium (MA^+^) ions are common (over 0.4% at room temperature) and dominate diffusion mechanisms. Ion migration primarily occurs *via* vacancy-assisted diffusion, with X-site anions (I^−^) showing the highest mobility (activation energy ∼0.58 eV), A-site cations (MA^+^) having moderate mobility (∼0.84 eV), and B-site cations (Pb^2+^) being largely immobile due to high migration barriers (∼2.31 eV), contributing to the structural stability of the perovskite lattice.^41,42^Fig. 6 illustrates the schematic diagram of vacancy diffusion-translocation mechanism for halide exchange reaction.

**Fig. 6:**
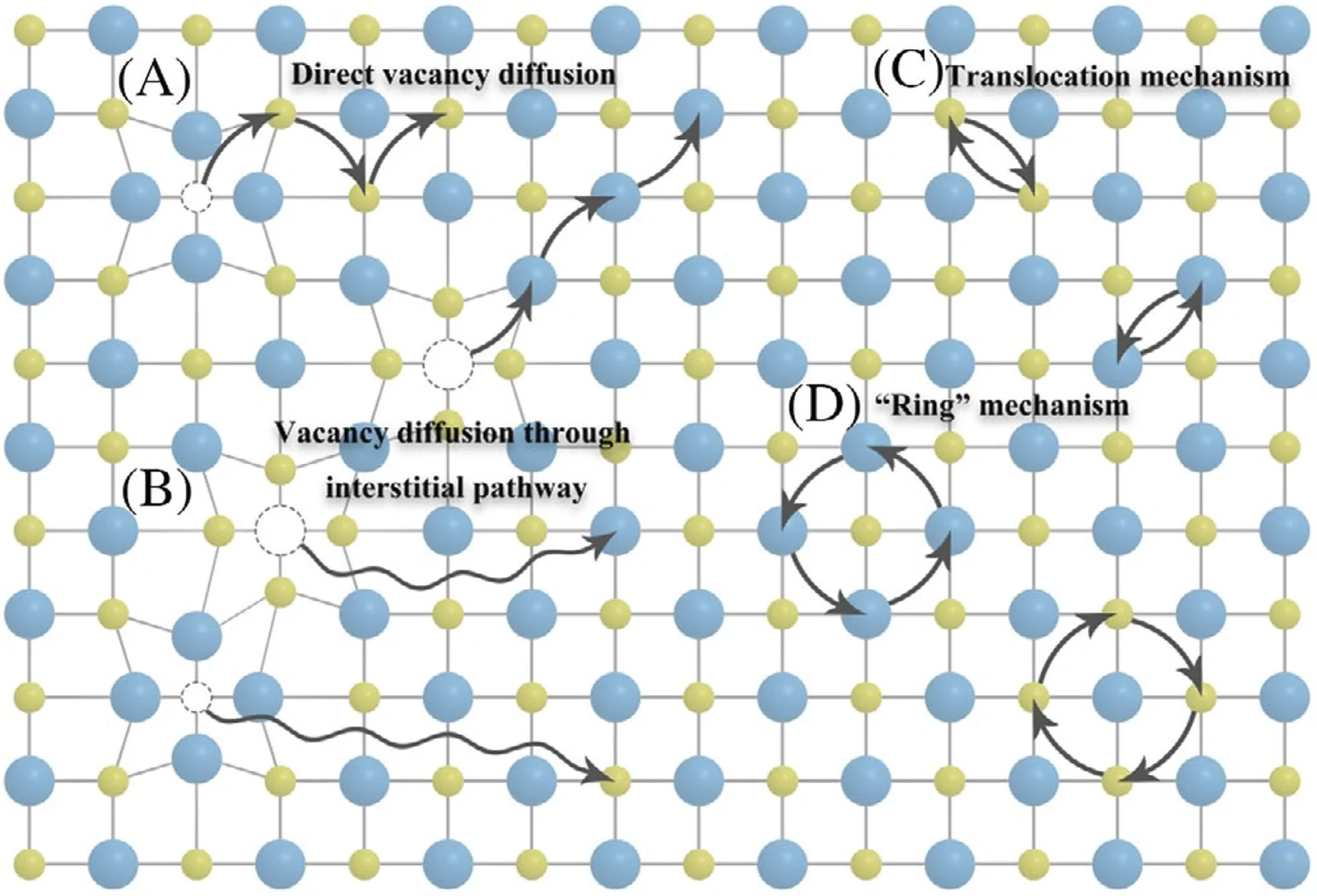
Schematic illustration of distinct atomic-scale ion migration and diffusion pathways within a hybrid perovskite crystal lattice of MAPbX_3_. (A) Direct vacancy diffusion, where an adjacent lattice ion migrates straight into an open vacant site. (B) Vacancy diffusion through an interstitial pathway, depicting complex migration hops bypassing normal lattice positions *via* interstitial spaces. (C) Translocation mechanism, showing a concerted exchange or localized structural shift between neighboring atomic sites. (D) “Ring” mechanism, demonstrating a coordinated, multi-atom rotational exchange loop that leaves no net permanent vacancies.^41^ Reproduced from ref. 41 with permission from [Willey] [*Nano Select*], copyright 2026.

## 5. Strategies for spatial control of halide exchange

### 5.1 Comparative framework for spatial control

Spatial control can be achieved by controlling either the location of the halide source, the local chemical driving force, the transport pathway, or the reaction time. These control axes lead to distinct trade-offs. Laser-assisted and photocatalytic approaches provide direct optical addressability and can generate localized emission within individual crystals or nanostructures, but the patterned area and throughput are generally limited. Lithographic masking improves geometric definition by restricting the exchange reagent to predefined openings, although subsequent halide interdiffusion can broaden the compositional boundary. Solution–solid and solid–solid diffusion are comparatively simple and can generate extended gradients, but their spatial resolution is governed by diffusion length and reaction time. Vapor-phase exchange can address patterned films or individual nanoplates with controlled exposure, while offering a route toward larger-area processing. Microfluidic delivery is best viewed as an enabling platform for controlling precursor transport and reaction uniformity; the present literature provides stronger evidence for its potential to improve scalability than for a standardized, quantitatively benchmarked spatial resolution. Across all approaches, the key performance metric is therefore not merely whether exchange occurs, but whether the final composition remains confined to the intended region.

For clarity, the quantitative comparison in Table 2 uses “NR” where the reviewed source does not report a numerical value. This distinction is important because several studies demonstrate spatially resolved photoluminescence but do not directly report the chemical boundary width. PL maps are therefore treated as evidence of spatial functionality, whereas EDS/XPS or equivalent compositional mapping provides stronger evidence of chemical localization.

**Table 2:** Quantitative comparison of representative spatially controlled halide-exchange strategies. “NR” = not reported in the reviewed source

Material/morphology	Exchange phase	Halide source	Localization method	Spatial resolution	Processing time	Spectral shift/outcome	Stability evidence	Main limitation
CsPbBr_3_ nanocrystals^30^	Liquid–solid/light-mediated	Haloalkanes; Br → Cl/I	Photocatalytic/light patterning	Nanocrystal scale; exact feature width NR	Minutes; exact value NR	Visible-range multicolor PL	Pattern stability duration NR	Surface chemistry and spatial confinement
MAPbBr_3_ single crystal (20 × 20 µm^2^)^53^	Liquid–solid	MAI (300 µM)	1064 nm NIR laser trapping	Localized µm-scale region; exact width NR	≈1 min bulk exchange; 30 min localized irradiation	Green (∼549 nm) → red; MAPb(Br_*x*_I_1−*x*_)_3_	Long-term stability NR	Bulk exchange competes with local confinement
MAPbBr_3_ microrod^54–56^	Liquid–solid	MAI	1064 nm, 0.5 W CW NIR laser	Localized end/central regions; exact width NR	Tens of minutes	Green (∼540 nm) → red (∼750 nm)	Long-term stability NR	Diffusion broadens the localized region
MAPbBr_3_ microrod^56^	Liquid–solid	MABr followed by MAI	Laser-assisted vacancy filling	Central inhibited region; exact width NR	20 min MABr treatment before MAI	Green region retained; exchange suppressed locally	Long-term stability NR	Requires controlled vacancy population
CsPbBr_3_ nanowire^57^	Liquid–solid/masked exchange	Oleylammonium chloride/iodide	EBL-defined PMMA opening	µm-scale; 10 µm image scale bar; exact feature width NR	NR	Green → blue (Br/Cl) or green → NIR (Br/I) gradients	Spatial PL retained after 1 week in N_2_ in representative nanowires	Interdiffusion broadens interfaces; EBL is low-throughput
CsPbBr_3_ thin film/gradient-bandgap film^58^	Solution–solid	CsI or CsCl	Controlled dipping/withdrawal	Gradient across film; exact resolution NR	Controlled by dipping speed; exact exchange time NR	Continuous multicolor emission gradient	Long-term stability NR	Diffusion limits sharp spatial boundaries
CsPbBr_3_/CsPbCl_3_ nanowire heterostructure^59^	Solid–solid	Adjacent CsPbCl_3_/CsPbBr_3_	Direct interfacial contact	Gradient length tunable; exact width NR	Several days at room temperature	Deep blue → green spatial PL gradient	Long-term stability NR	Slow kinetics and spontaneous diffusion
CsPbBr_3_/δ-CsPbI_3_ nanowire crossbar^60^	Solid–solid	δ-CsPbI_3_ source-limited iodine	Spatially constrained crossbar source	Tens of µm gradient reported	20–90 min; 200–240 °C	Green → yellow → red → NIR gradient	Long-term stability NR	Elevated temperature and diffusion control required
CsPbBr_3_ nanoplates^65^	Vapor–solid	*n*-C_4_H_9_NH_3_I vapor	Localized vapor exposure/geometry-dependent exchange	Thickness-dependent 20–380 nm regions analyzed	10–25 min	Green PL → iodine-rich longer-wavelength emission	Long-term stability NR	Thickness/edge effects and vapor control
Patterned MAPbI_3_/MAPbBr_3_ thin films^66^	Vapor-phase	MAI followed by MABr	Pre-patterned lines + *in situ* exchange	µm-scale patterned lines; exact width NR	10–30 min MABr exposure	I-rich → Br-rich spectral evolution	Long-term stability NR	Pattern fidelity *versus* vapor diffusion

While bulk halide exchange enables overall bandgap tuning, it lacks control over localized emission regions essential for high-resolution optoelectronics. To address this, several innovative strategies have emerged to enable spatially confined halide exchange reactions in hybrid perovskites. These reactions can occur in solid–solid,^43,44^ liquid–solid,^22^ or gas–solid^45^ phases, and are facilitated by advanced techniques. Laser trapping, for instance, offers precise control over localized areas within a crystal, enabling selective exposure to specific halide exchange solutions.^46^ Patterning techniques such as photolithography can also be used to define specific regions of a perovskite film for targeted halide exchange reactions.^47^ In addition to sophisticated techniques, more traditional methods like temperature control and photocatalysis also hold promise for halide exchange in perovskites. Temperature manipulation can influence the kinetics of the exchange reaction, while photocatalysis can provide an alternative pathway for the process. These approaches offer potential advantages in terms of simplicity and scalability, making them attractive for certain applications. By exploring these and other innovative approaches, researchers aim to achieve precise control over halide composition at various spatial scales.

## 6. Photocatalytic assisted spatial halide exchange in hybrid perovskite: UV light controlled

Light-driven spatial halide exchange techniques, such as photocatalytic halide exchange, utilize spectral control and chemical reactivity for precise ion migration in perovskite nanocrystals. In this approach, light acts as both the trigger and the guide: photocatalytic reactions harness photon energy to activate the cleavage of carbon–halogen bonds, enabling efficient surface-limited halide exchange as demonstrated with cesium lead halide perovskite nanocrystals. Molecular haloalkanes are introduced to control the halide composition within the nanocrystal lattice, allowing for complete visible spectrum tuning and the creation of photoluminescent trichromatic patterns—an advance for display technology.^22^ The experimental setup involves synthesizing CsPbBr_3_ nanocrystals dispersed in dichloromethane, where the use of thiophenol enhances photoluminescence. Upon white LED illumination, the halide exchange process is initiated, resulting in gradual blueshifts in emission as Br^−^ ions are replaced by Cl^−^. This results is shown in the Fig. 7. This photo-activated process exemplifies surface-mediated chemistry. While photocatalytic halide exchange leverages light-triggered, spatially selective reactions, techniques like laser trapping provide even finer mechanical and spatial precision for localized halide exchange within individual crystals.^22^

**Fig. 7:**
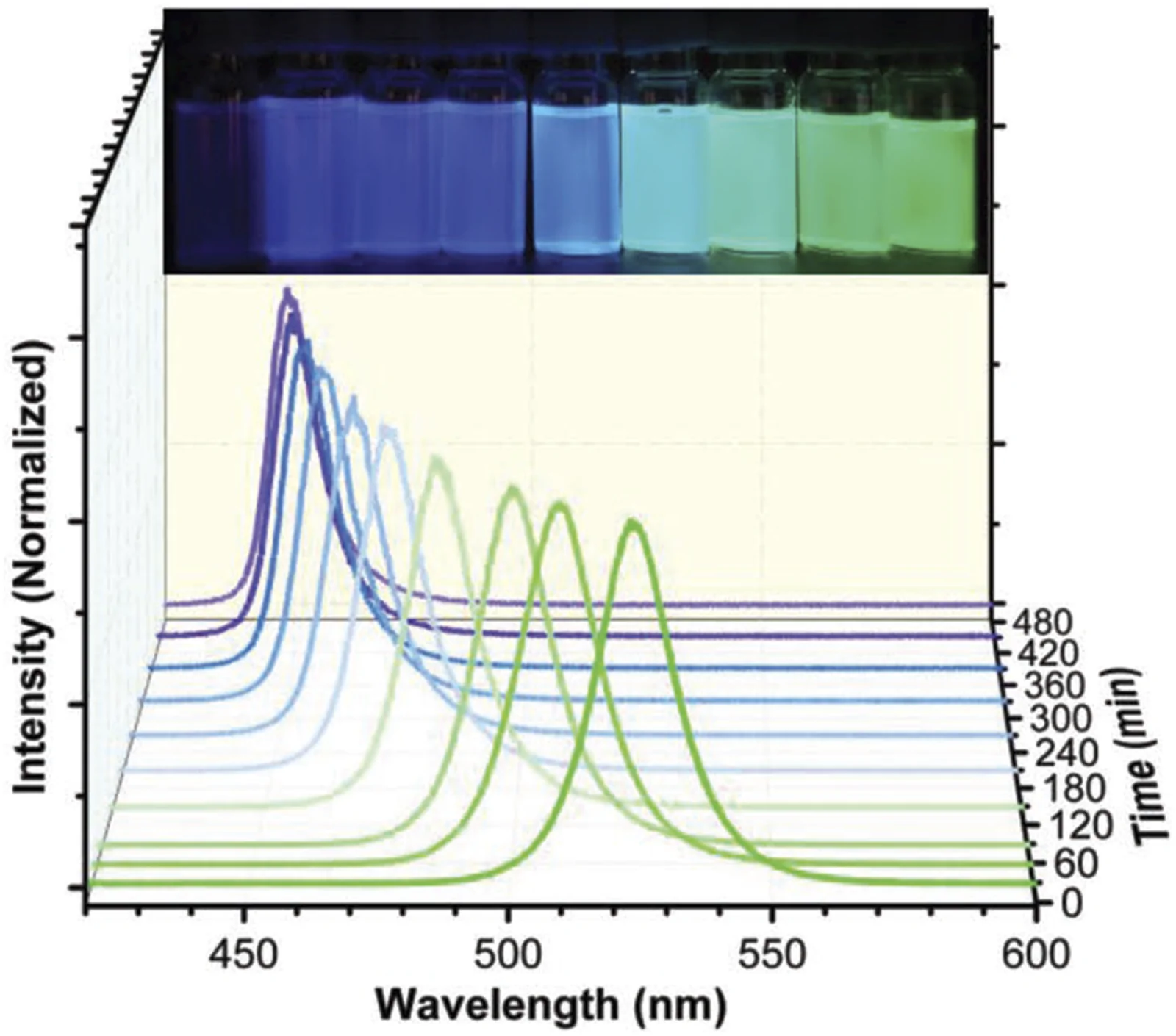
PL spectra of CsPbBr_3_ nanocrystals in DCM show emission shifts, with visible photoluminescence under 365 nm UV.^30^ Reproduced from ref. 30 with permission from [Willey] [*Advanced Materials*], copyright 2026.

## 7. Laser trapping induced approaches for spatial halide exchange reaction

Laser trapping technique utilizes a tightly focused laser beam to manipulate microscopic objects. In the context of halide exchange, the laser beam can be used to trap a specific region of a perovskite crystal while simultaneously exposing it to a solution containing the desired replacement halide ions. This localized irradiation facilitates the exchange process only within the trapped area, leaving the surrounding perovskite structure untouched. For instance, researchers have employed laser trapping to achieve spatially selective iodination within a methylammonium lead bromide (MAPbBr_3_) perovskite crystal.^47^

These light-mediated techniques offer several advantages. They are non-contact, reducing the risk of physical damage to the perovskite film during processing.^47^ Additionally, the high spatial resolution afforded by focused light enables precise control over patterning, which is crucial for the fabrication of complex micro- and nanoscale emission domains.^5^ Laser trapping induced spatial halide exchange in bulk perovskite.

To achieve localized emission color tuning, a focused 1064 nm near-infrared (NIR) laser was employed to target specific regions of the MAPbBr_3_ crystal in the presence of the MAI solution. This optical gradient force induced the selective incorporation of iodide ions at the irradiated site, forming MAPb(Br_*x*_I_1−*x*_)_3_ with a red-shifted emission localized within the green-emitting host. Post-irradiation photoluminescence imaging revealed a spatially resolved green-to-red emission transition—evidence of successful halide exchange at the targeted region. Spectroscopic analysis further confirmed this effect, marking a clear shift in the emission spectrum and supporting the concept of optically addressable color patterning within individual perovskite microcrystals. Here the crystals were fabricated using the solvent evaporation method, where a microdroplet of MAPbBr_3_ precursor solution was deposited on a glass substrate and allowed to evaporate, resulting in the formation of 20 × 20 µm crystals. Characterization through transmission microscopy and emission spectroscopy confirmed the formation of MAPbBr_3_ perovskite with a characteristic green emission peak at 549 nm.^47^ To induce spatial emission color changes, a 300 µM MAI reaction solution was introduced into the reaction chamber containing the MAPbBr_3_ crystal. A bulk emission color change from green to red was observed within 1 minute. However, to achieve localized emission color modification, a focused laser was used to target a specific area of the crystal. This resulted in the formation of a red-emitting region within the green-emitting crystal. The red-shifted emission is a direct consequence of iodide incorporation driven by the laser's optical gradient force, which concentrates the MAI precursor at the targeted region. Spectroscopic analysis confirmed the incorporation of iodide ions into the targeted area, leading to a red shift in the emission band and the formation of MAPbBr_2.3_I_0.7_ perovskite composition. The color modification results are illustrated in the Fig. 8.

**Fig. 8:**
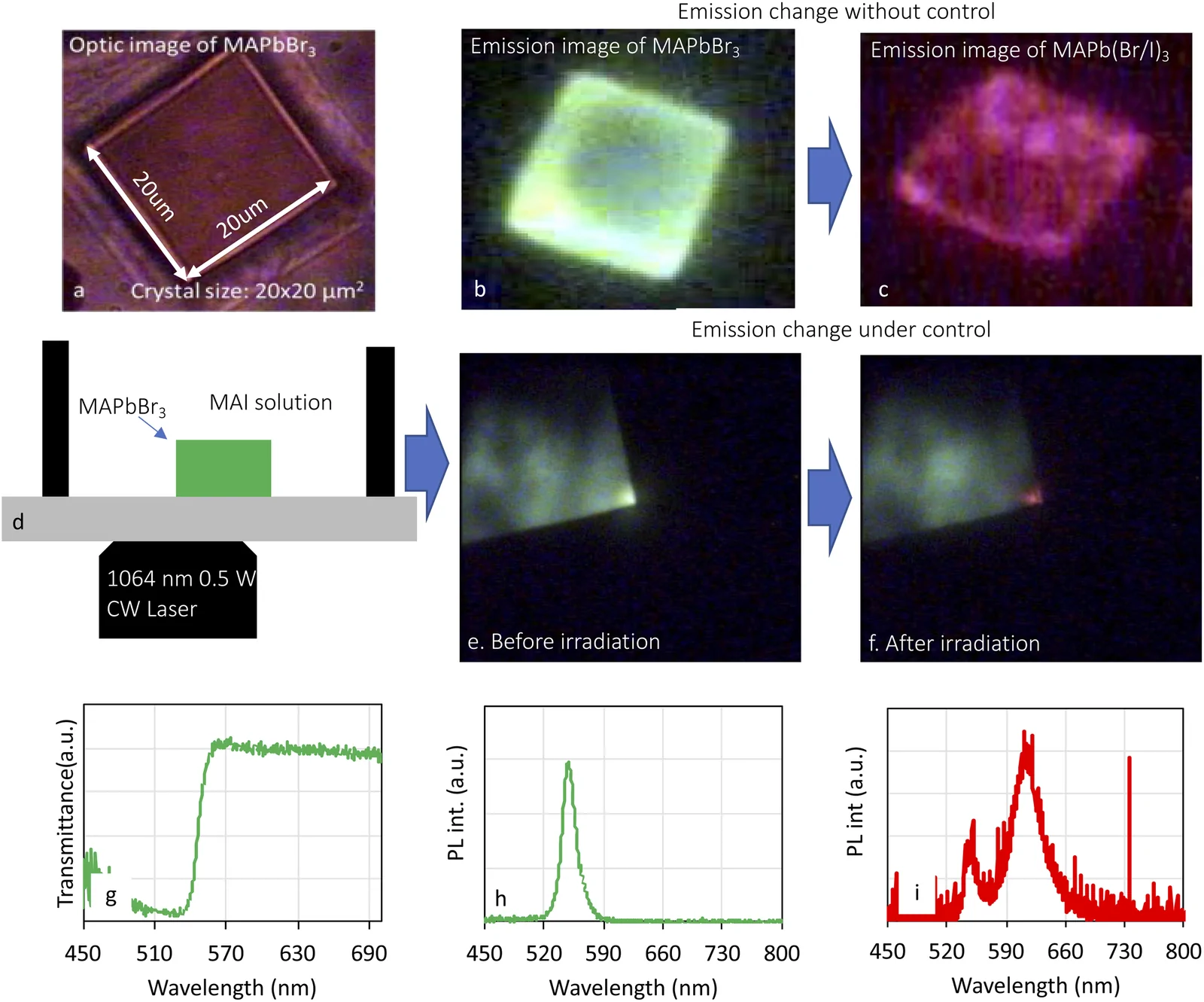
Micro-spectroscopic analysis and laser-induced spatial emission control in MAPbBr_3_ perovskite microcrystals. (a) Optical transmission micrograph of a synthesized MAPbBr_3_ single crystal (dimensions: 20 × 20 µm^2^) *via* solvent evaporation from a MABr + PbBr_2_/DMF precursor solution. (b) Characteristic uniform green photoluminescence (PL) emission image of the pristine MAPbBr_3_ microcrystal before halide exchange. (c) Red PL emission image from the mixed-halide MAPb(Br/I)_3_ phase following controlled anion exchange. (d) Schematic of the experimental setup utilizing a 1064 nm, 0.5 W continuous wave (CW) laser to assert optical forces and localized heating. (e and f) Wide-field PL emission images captured (e) before and (f) after a 30-minute near-infrared (NIR) laser irradiation sequence, showing localized red-shifting at the targeted corner vertex. (g) Optical transmittance spectrum profile of the freshly synthesized pristine MAPbBr_3_ crystal. (h) Corresponding steady-state PL emission spectrum of pristine MAPbBr_3_ centered in the green spectral window. (i) Multi-peak local PL emission spectrum of the modified MAPb(Br/I)_3_ crystal after 30 minutes of targeted NIR laser irradiation.^47^ Reproduced from ref. 47 with permission from [Elsevier] [*Journal of molecular liquid*], copyright 2026.

Fig. 9 and 10 illustrate the photoluminescence (PL) behavior of a MAPbBr_3_ microrod crystal undergoing a localized halide exchange reaction by employing 1064 nm NIR laser of 0.5 W continuous-wave mode. In Fig. 9(a), the microrod exhibits uniform green emission under UV laser irradiation, characteristic of the MAPbBr_3_ composition. Following halide exchange with iodide, as shown in Fig. 9(b) through Fig. 9(d), a clear color change is observed at the ends of the microrod—from green to red—indicating successful conversion to MAPb(Br_*x*_I_1−*x*_)_3_. This spatially resolved halide exchange is further validated by the PL spectrum in Fig. 9(e), where the green emission peak (∼550 nm) of the non-irradiated region contrasts sharply with the red-shifted peak (∼750 nm) from the iodide-rich irradiated region. Fig. 9(d) labels these distinct regions as MAPbBr_3_ (green) and MAPb(Br_*x*_I_1−*x*_)_3_ (red), confirming that the 1064 nm NIR laser exposure effectively drives a controlled halide exchange, enabling site-specific tuning of the optical properties within a single microrod crystal.

**Fig. 9:**
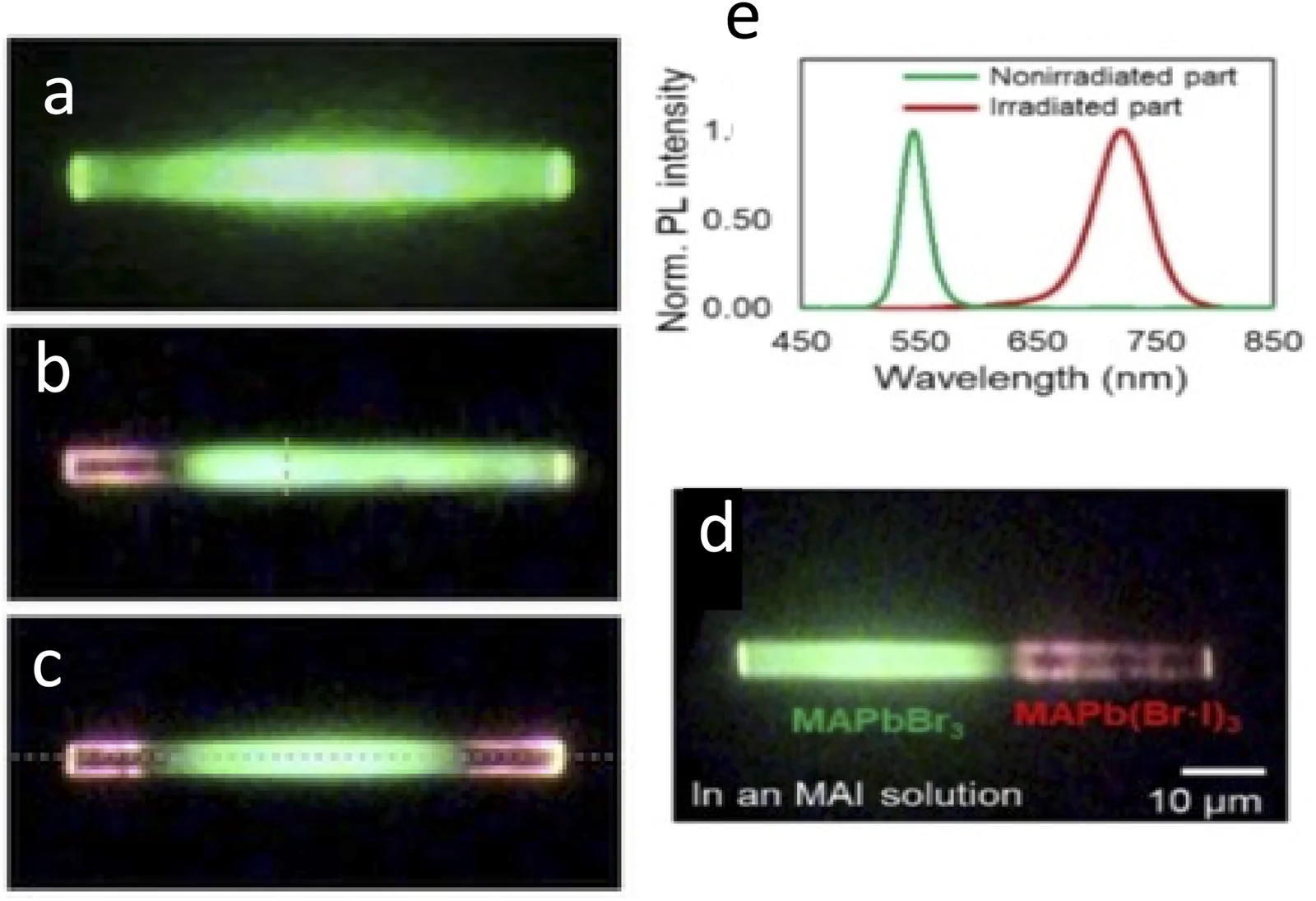
Spatial controlled halide exchange by employing 1064 nm NIR laser of 0.5 W continuous-wave mode by the reaction of MAPbBr_3_ + MAI = MAPb(Br_*x*_I_1−*x*_)_3_ (a) photoluminescence (PL) image of the microrod crystal under UV laser irradiation prior to the halide exchange reaction. (b–d) PL image of the same microrod crystal under UV laser irradiation following the halide exchange reaction (e) emission spectrum of MAPbBr_3_ crystal before and after halide exchange with iodide ion.^48^ Reproduced from ref. 48 with permission from [Wiley] [*Angewandte Chemie International Edition*], copyright 2026.

**Fig. 10:**
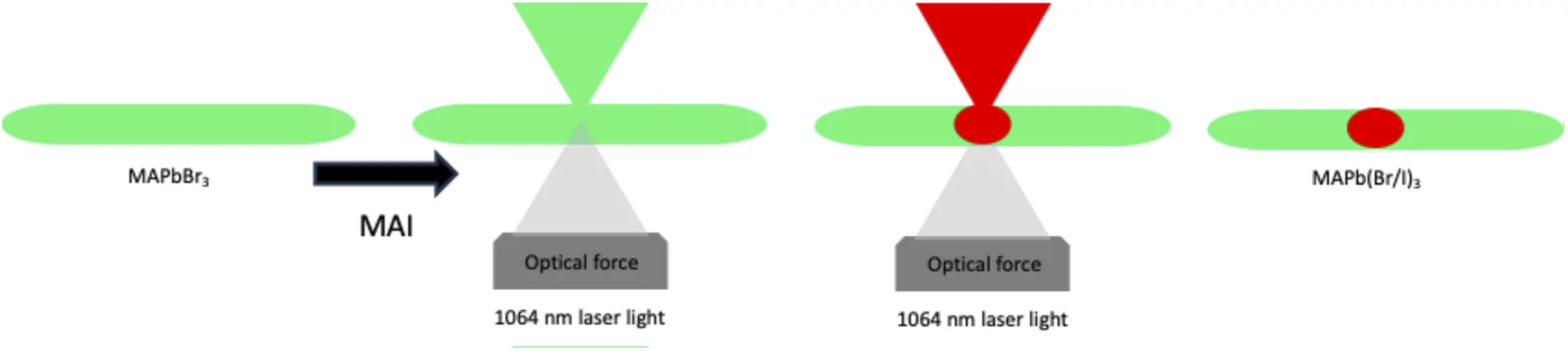
Mechanism of halide exchange in MAPbBr_3_ microplates and microrods *via* laser trapping.

The optical gradient force surpasses the random Brownian motion of MAI molecules, directing them toward the laser focus and creating a reaction hotspot. Within this concentrated zone, iodide ions (I^−^) efficiently replace bromide ions (Br^−^) in the perovskite lattice. The subsequent vacancy-assisted diffusion mechanism enables the incorporated iodide ions to propagate through the microstructure beyond the initial focal region.^47–50^ This diffusion is critical for converting larger areas of the MAPbBr_3_ crystal into a mixed-halide perovskite, such as MAPb(Br_*x*_I_1−*x*_)_3_, depending on exposure time and local conditions.

Laser trapping assisted selective area halogen exchange can also be demonstrated in another mechanism by suppression of halogen exchange in a predefined area which is demonstrated in the Fig. 11. For this purpose – a 250 µM MABr solution was introduced into the chamber (Fig. 11a(i)), and the microrod's center was then irradiated with a focused 1064 nm NIR laser beam (Fig. 11a(ii)).^50^ After 20 minutes, the laser was turned off, the MABr solution removed, and a 375 µM MAI solution was added to investigate bromide-to-iodide exchange (Fig. 11a(iii)). Fig. 11b shows PL images before and after MAI incubation. Initially, the MAPbBr_3_ microrod emitted green light across its entirety (Fig. 11b(i)). Following MAI incubation, the non-irradiated regions gradually shifted from green to red emission, indicating partial iodide incorporation into bromide vacancies. Critically, the laser-irradiated central area retained its green emission (Fig. 11b(ii)), demonstrating localized suppression of halide exchange due to NIR laser-assisted filling of Br vacancies. This preservation of the original emission is attributed to the laser-assisted filling of Br^−^ vacancies with excess MABr, which subsequently blocks iodide incorporation from MAI. PL spectra (Fig. 11c) in MAI solution revealed two peaks (545 nm and 615 nm) from the non-irradiated area, corresponding to MAPbBr_3_ and halide-exchanged MAPbBr_3−*x*_I_*x*,_ respectively. In contrast, the irradiated center showed only a 545 nm peak, similar to the initial crystal. This indicates that focused laser irradiation locally fills bromide vacancies in MAPbBr_3_ within a MABr solution, inhibiting subsequent bromide-to-iodide exchange in MAI solution.

**Fig. 11:**
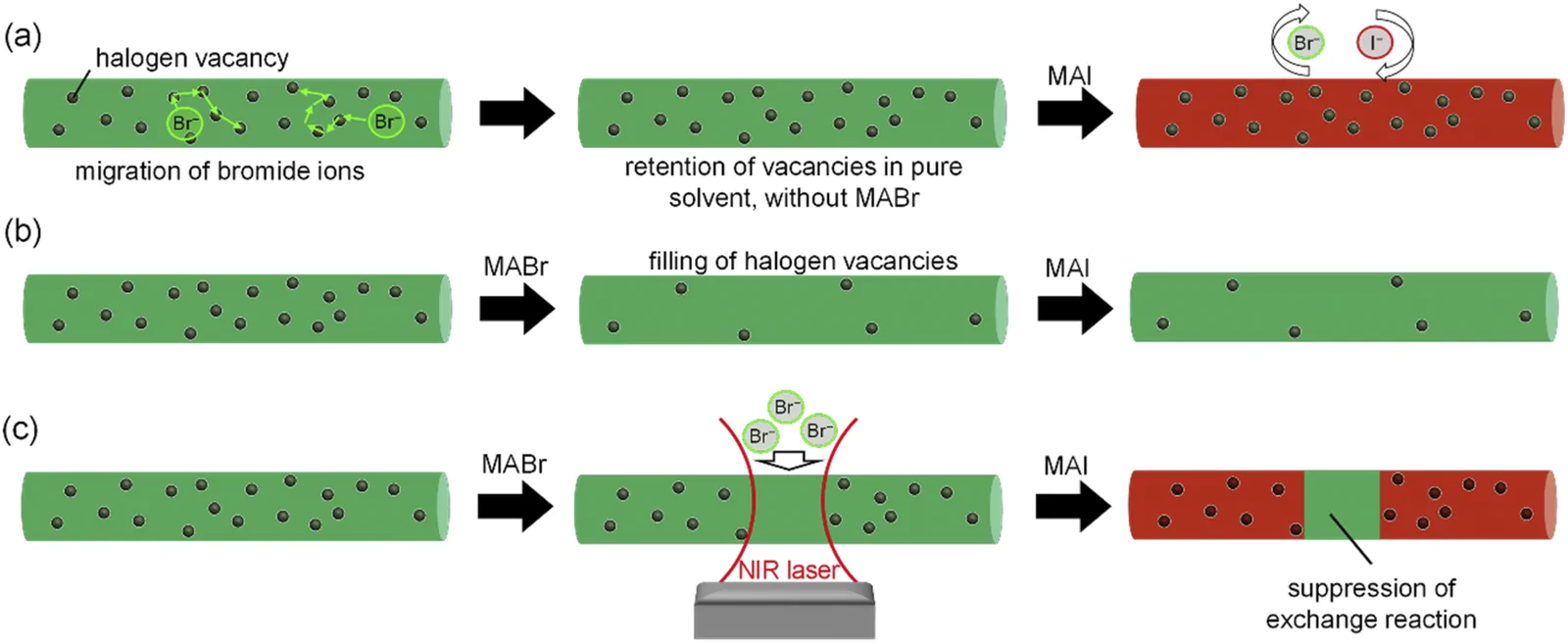
Schematic depicting of the suppression of chemically and optically controlled halide exchange in MAPbBr_3_ microrods through vacancy filling. (a) Microrod in pure solvent followed by MAI. (b) Microrod in MABr followed by MAI. (c) Microrod irradiated at the center in MABr followed by MAI.^50^ Reproduced from ref. 50 with permission from [Wiley] [*Advanced Materials Technologies*], copyright 2026.

## 8. Masking techniques by electron beam lithography for spatial halide exchange

Dou Letian, *et al.* demonstrated the fabrication of spatially defined cesium lead halide perovskite nanowire heterojunctions relies on a sophisticated marriage of electron-beam lithography (EBL) masking and highly controlled solution-phase or solid-state anion exchange.^51^ The structural integrity and precise positioning required for these low-dimensional optoelectronic devices are achieved by transferring pristine, high-aspect-ratio CsPbBr_3_ nanowires—synthesized *via* an inverse temperature crystallization method—onto a SiO_2_/Si platform. By spin-coating a protective poly(methyl methacrylate) (PMMA) resist layer over the wires and selectively carving out microscale windows *via* EBL, researchers create a localized chemical gateway. When exposed to reactive halide precursors, such as oleylammonium chloride or oleylammonium iodide dissolved in 1-octadecene (ODE), the exposed segment of the CsPbBr_3_ wire undergoes a thermodynamic, substitution-driven anion exchange, replacing the native bromide ions. Because the surrounding PMMA mask is strictly impermeable to the exchange solution, the conversion remains spatially restricted to the patterned opening. Upon stripping the resist with orthogonal solvents like chlorobenzene and hexanes, an intact single-nanowire heterojunction is revealed. The experimental protocol and consequential results are illustrated in the Fig. 12. The true power of this selective-exposure technique lies in the kinetics of the subsequent interdiffusion process at the junction boundary. Rather than forming atomically abrupt interfaces, which are often prone to high defect densities and non-radiative recombination centers, the solid-state diffusion of halide ions yields smooth, texturally continuous compositional gradients over several micrometers. As observed under focused laser excitation, these continuous variations manifest as spatially shifting photoluminescence (PL) profiles—transitioning seamlessly from green to blue for partial Br/Cl exchanges, and from green to near-infrared for Br/I variations. This continuous structural adjustment smoothly shifts the semiconductor's electronic energy bands along the length of a single wire. Consequently, this masking technique provides a reliable path toward complex, single-nanowire optoelectronic architectures.

**Fig. 12:**
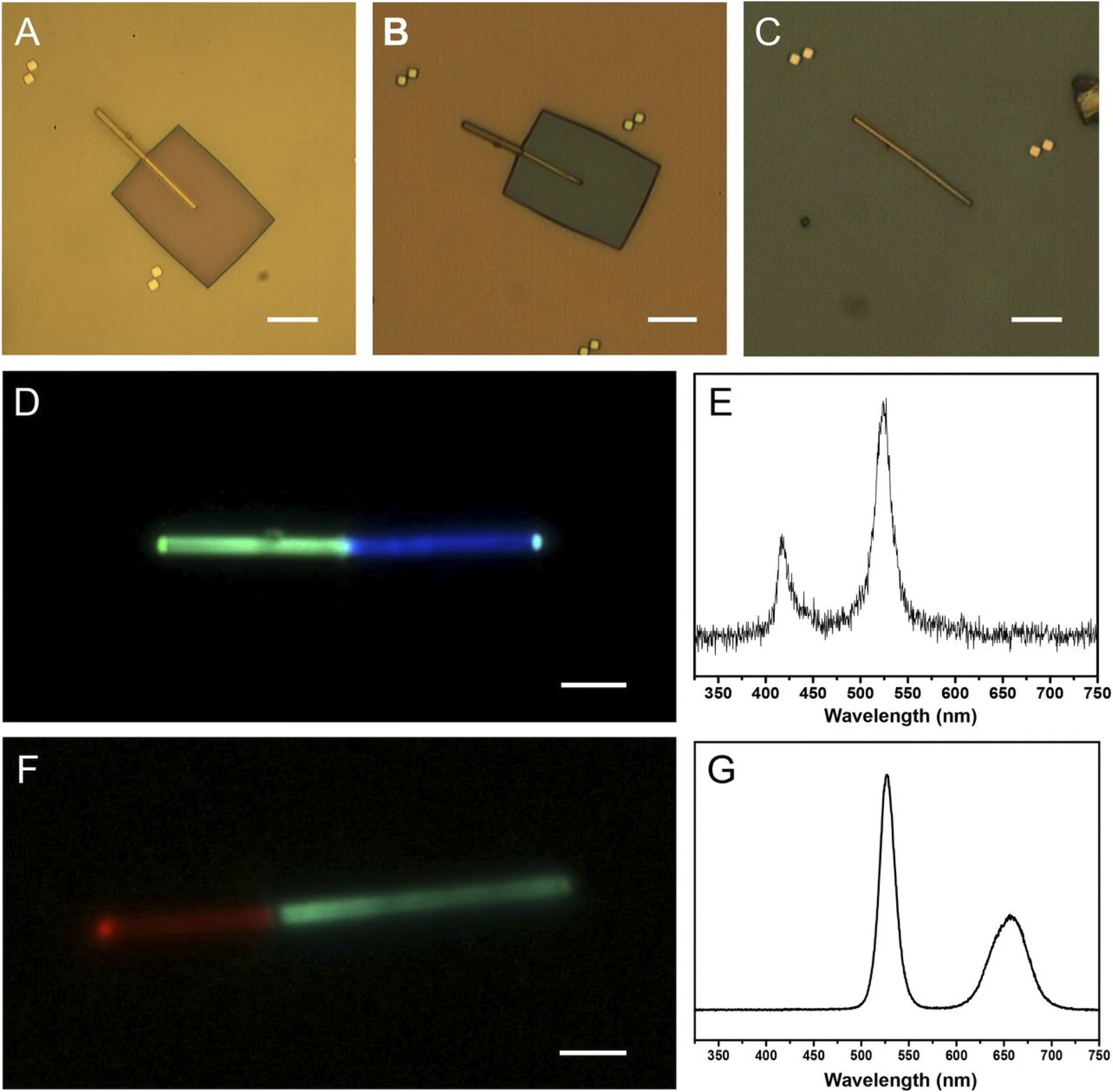
Fabrication and optical characterization of compositionally graded cesium lead halide perovskite nanowire heterojunctions. (A–C) Spatial profiling sequence showing a single CsPbBr_3_ nanowire masked by a PMMA layer with an EBL-patterned square window before anion exchange (A), following localized solid-state halide conversion (B), and after PMMA template removal (C) (scale bars: 10 µm). (D and F) Cross-sectional photoluminescence (PL) micrographs under focused laser excitation visualizing smooth emission profiles along partial Br/Cl (D) and Br/I (F) interdiffusion gradients (scale bars: 3 µm). (E and G) Corresponding steady-state PL emission spectra highlighting the precise, position-dependent bandgap tuning achieved across the heterojunction axes.^51^ Reproduced from ref. 51 with permission from [PNAS] [*Proc. Natl. Acad. Sci. U. S. A.*], copyright 2026.

## 9. Concentration gradient force implied halide exchange

One established technique for halide exchange in hybrid perovskites is gradient-based exchange *via* diffusion. Zhang Mei-Na, *et al.* successfully applied this technique to convert green-emitting bromide-based perovskites into red-emitting iodide-based counterparts.^52^ In-fact this method relies on the inherent tendency of molecules to move from areas of high concentration to low concentration. In this process a pristine perovskite film is placed in contact with a solution containing the desired halide ions (*e.g.*, replacing chlorine with bromine). A concentration gradient is established across the perovskite film, with the highest concentration of the incoming halide at the interface and gradually decreasing towards the interior. This gradient drives the diffusion of the incoming halide ions into the perovskite lattice, replacing the original halide ions through an exchange process. The rate of exchange can be controlled by factors like the initial concentration difference, temperature, and diffusion time. Fig. 13 illustrates the process of creating perovskite films with a gradient in their bandgap (and thus emission color), referred to as “rainbow” films, and the characterization of these films. A perovskite film is dipped into a solution containing a different halide, causing an exchange of halide ions within the film.^52^ The speed at which the film is dipped and withdrawn influences the extent of the halide exchange. Fluorescence microscopy images of Br/I gradient bandgap (GBG) films created using different dipping speeds (1–5 mm s^−1^) in a 16 g per L CsI solution are shown.^52^

**Fig. 13:**
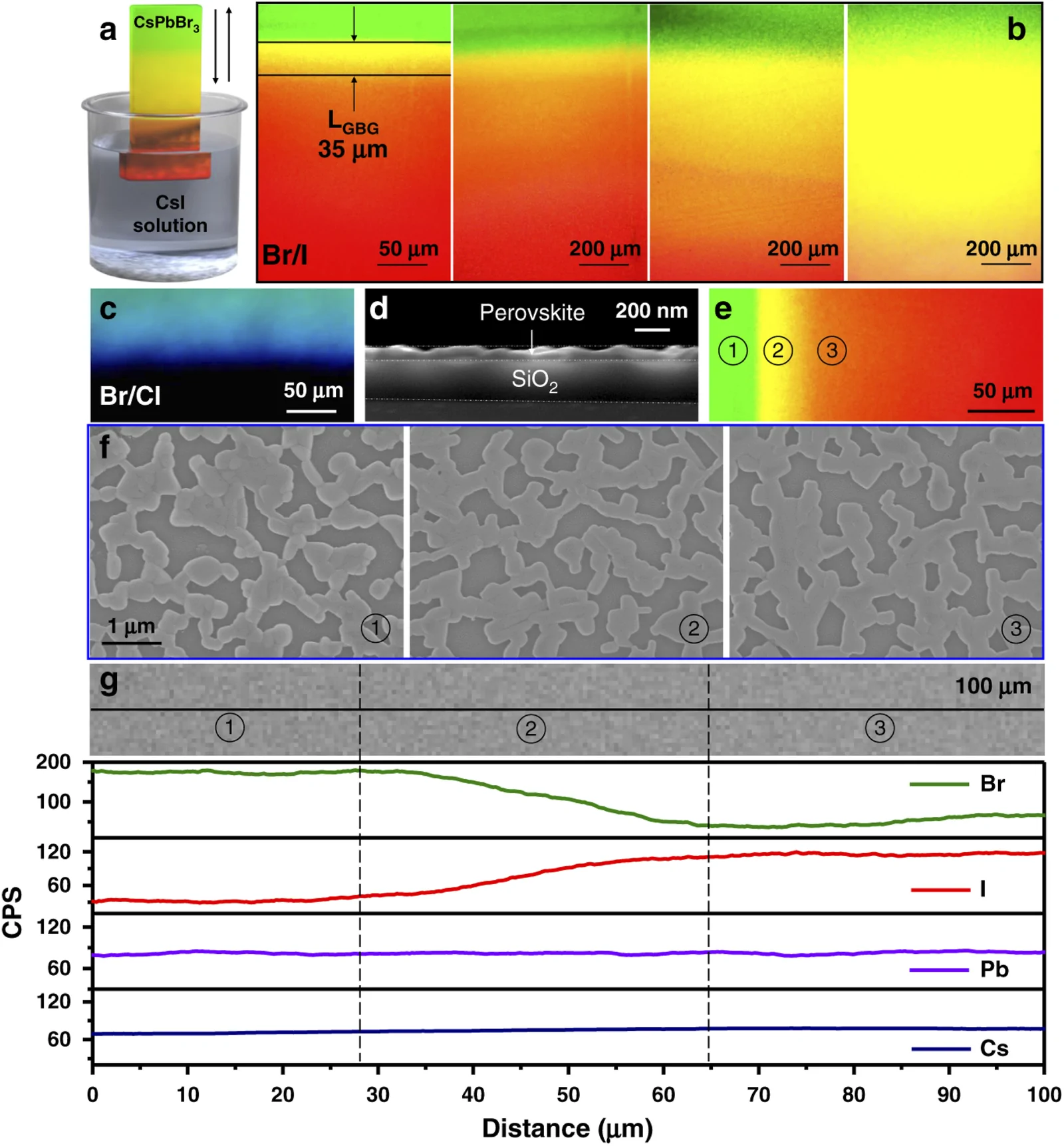
Rainbow perovskite films fabrication and analysis. (a) Dipping method for halide exchange. (b) Fluorescence microscopy of Br/I gradient bandgap (GBG) films (dipping speeds 1–5 mm s^−1^, 16 g per L CsI) and (c) Br/Cl GBG film (1 mm s^−1^, 16 g per L CsCl). (d) Cross-sectional SEM of a Br/I GBG film. (e) Points of interest on a Br/I GBG film, (f) SEM images and (g) elemental line scans.^52^ Reproduced from ref. 52 with permission from [Springer Nature] [*Light: Science & Applications*], copyright 2026.

## 10. Solid–solid phase halide exchange by concentration gradient force

To investigate the halide ion diffusion process, we initiated our study by examining the chloride–bromide interdiffusion within a CsPbCl_3_/CsPbBr_3_ heterostructure reported by Pan Dongxu, *et al.*^53^ On that study results are illustrated in the Fig. 14. Fig. 14A presents a visual representation of the as-fabricated heterostructure, showcasing its layered architecture.^54^ The accompanying SEM image (Fig. 14B inset) provides microscopic evidence of the intimate physical contact between the two perovskite layers, a crucial prerequisite for efficient solid-state interdiffusion. This direct contact facilitates the exchange of halide ions across the interface, enabling the formation of mixed halide perovskites within the heterostructure.^55^ By maintaining contact between solid state CsPbBr_3_ NWs and CsPbCl_3_ microplates at room temperature, it was observed the spontaneous formation of heterojunctions with varying halide compositions within a few days. This spatial PL variation stems directly from the halide composition gradient formed by interdiffusion at the CsPbCl_3_/CsPbBr_3_ interface, as further confirmed by EDS elemental mapping, Fig. 14F–H. The resulting NWs exhibit distinct spatial variations in photoluminescence (PL) emission, spanning from deep blue to green, indicative of a continuous halide composition gradient from Cl-rich to Br-rich regions, Fig. 14C–E. This gradient formation is attributed to the rapid diffusion of halide ions within the perovskite lattice at room temperature. Energy-dispersive spectroscopy (EDS) analysis further confirms the compositional gradient, with decreasing Cl concentration and increasing Br concentration from the contact region to the end of the NW. The length of the gradient region can be controlled by adjusting reaction parameters such as temperature and time. To comprehend the kinetics of the halide exchange process, a thorough understanding of its underlying mechanism is essential. The proposed mechanism involves the formation of a “reaction zone” at the interface between the CsPbCl_3_ microplate and CsPbBr_3_ nanowire (NW). This zone facilitates rapid halide ion (Br^−^ and Cl^−^) exchange due to the abrupt concentration gradient. Subsequently, the exchange reaction progresses along the NW *via* solid-state ion diffusion, with Cl^−^ and Br^−^ ions moving in opposite directions. This mechanism bears resemblance to the extensively studied cation interdiffusion phenomena observed in transition metal chalcogenides and oxides.^56–58^ The mechanism of halide ion is mediated by diffusion within perovskite nanowires (NWs), specifically focusing on CsPbBr_3−*x*_Cl_*x*_. It proposes a vacancy-assisted diffusion mechanism for the solid-state ion exchange process, supported by previous research indicating high vacancy concentrations in halide perovskite.^59^ To study the diffusion kinetics in real-time, the authors utilize photoluminescence (PL) imaging to monitor the structural transformation within the gradient NWs. The evolution of emission color, directly correlated with the changing halide composition, allows for tracking the diffusion front. The linear relationship between the diffusion length and the square root of reaction time confirms the diffusion-controlled nature of the process, consistent with diffusion theories.

**Fig. 14:**
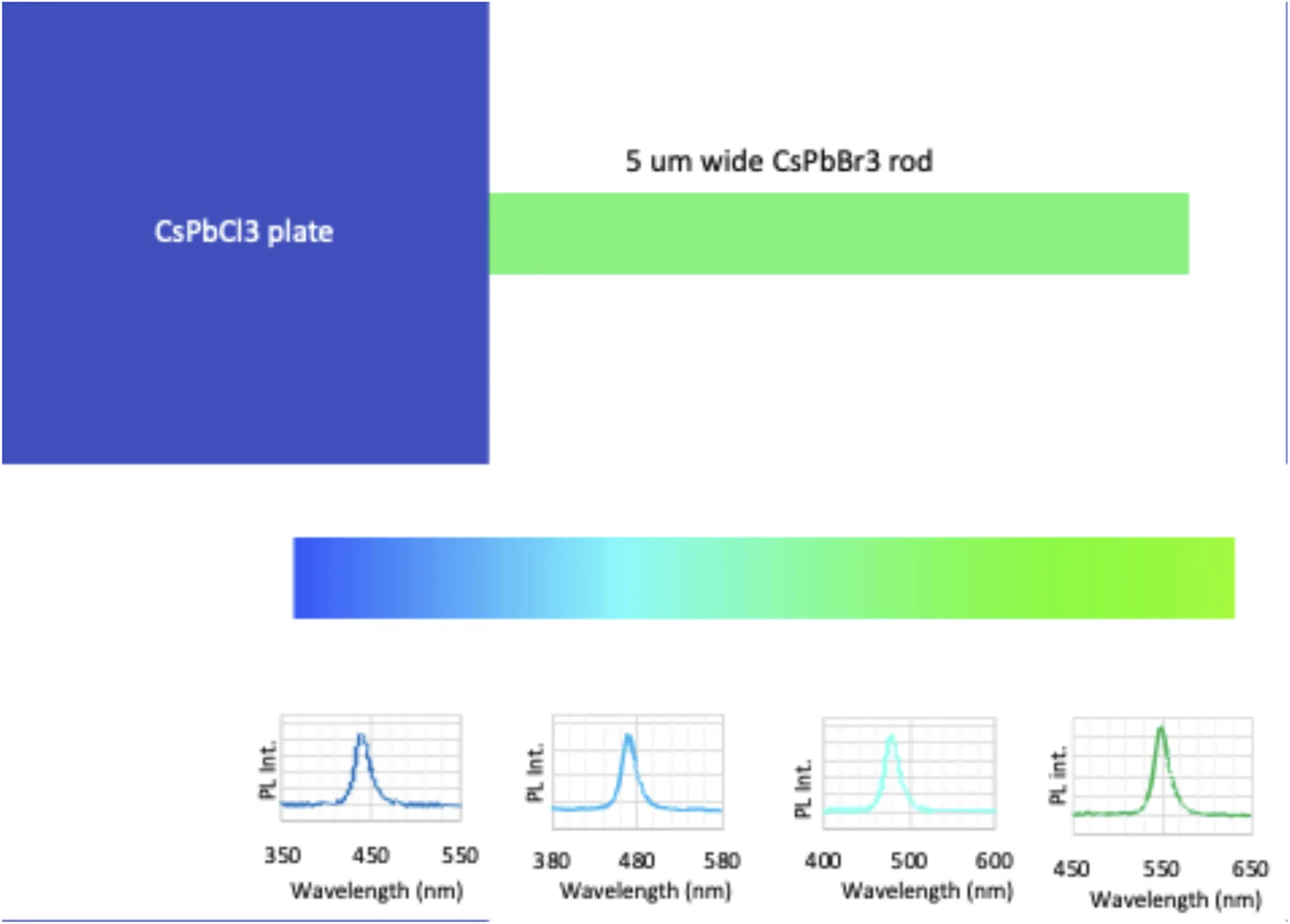
Halide exchange and compositional gradient formation in CsPbBr_3_/CsPbCl_3_ heterostructures. Schematic illustration of the interfacial halide exchange and subsequent axial Cl/Br interdiffusion. Optical emission images showing color change due to Cl–Br gradient in nanowires.

Another solid–solid state reaction for halide exchange in hybrid perovskite is reported by the Li Jing, *et al.*^60^ That anion exchange procedure begins with the formation of a microscale crossbar heterostructure composed of intersecting CsPbBr_3_ and δ-CsPbI_3_ nanowires. The results are shown in the Fig. 15. As shown in the spatial overlap between the individual nanowires creates a distinct intersection point with an intact, single-crystalline morphology and sharp, well-defined boundaries. Using the δ-CsPbI_3_ nanowire—which features one-dimensional anionic channels—as a source-limiting agent for iodine, the solid-phase anion exchange reaction is performed across a series of temporal intervals (20, 30, 40, and 90 minutes) and elevated temperatures (200 °C, 220 °C, and 240 °C). Optical and photoluminescence (PL) microscope tracking reveals that as reaction time increases at a constant temperature of 220 °C, the initially green-emitting CsPbBr_3_ wire incorporates iodine propagating outward from the crossbar junction.^61^ This diffusion gradually converts the local emission profile into a bright yellow, red, and near-infrared gradient, visually corresponding to a localized decrease in the semiconductor bandgap. Crucially, the gradient profile can be precisely controlled by modulating both the exchange duration and thermal energy. At lower temperatures (200 °C), the exchange is restricted to regions near the junction point, whereas higher temperatures (240 °C) accelerate the rate of diffusion along the axis. The significant finding of this observation is that by using a spatially constrained solid-state precursor to limit the local abundance of available halide ions, the typical rapid and random volatility of liquid- or gas-phase anion exchanges is overcome.

**Fig. 15:**
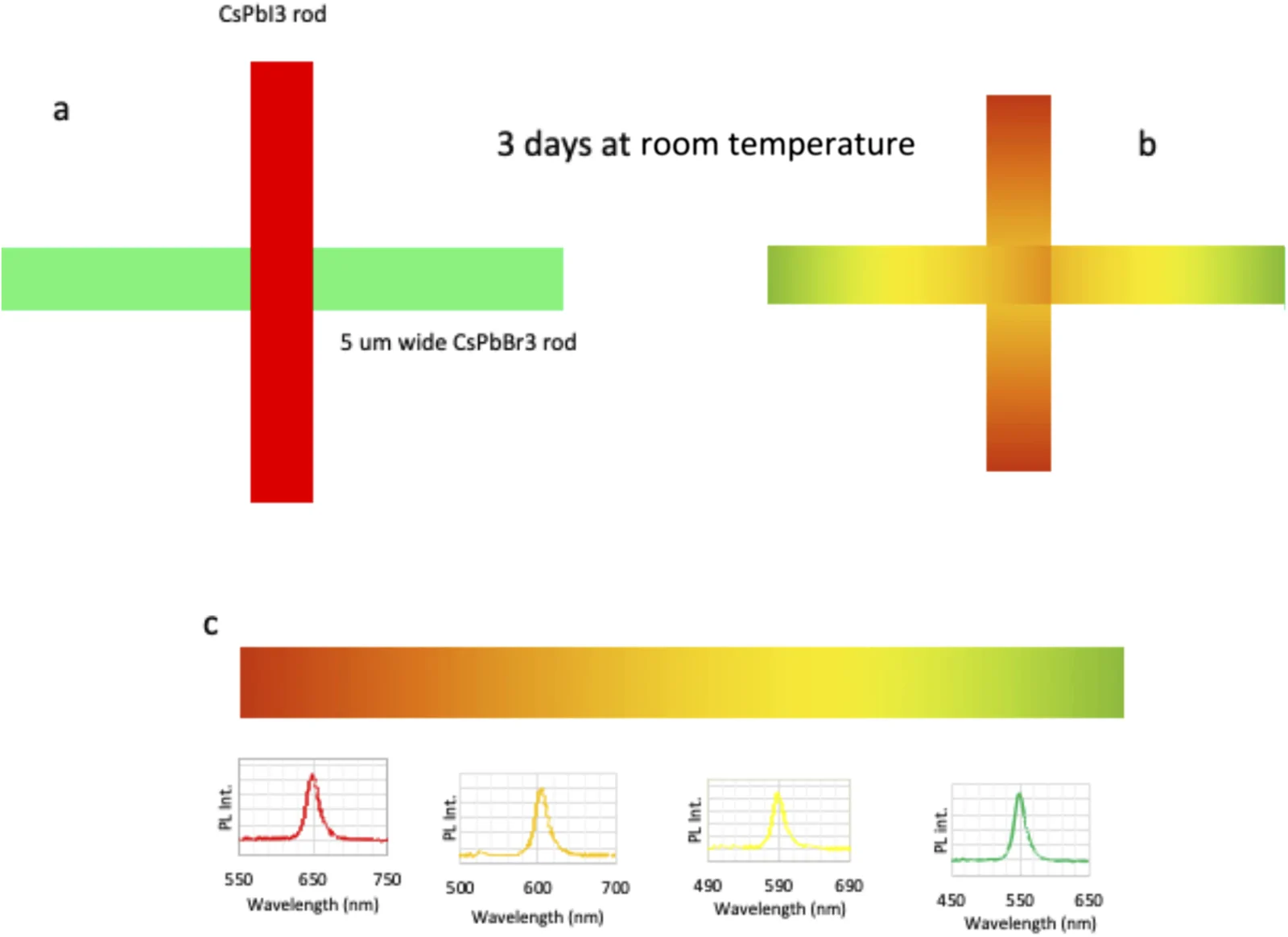
Structural and morphological characterization of perovskite crossbar heterostructures. (a–c) Time-resolved optical microscope images of the CsPbBr_3_ and δ-CsPbI_3_ nanowire crossbar junctions undergoing localized halide exchange at a constant temperature of 220 °C for 20 min (a), 30 min (b), 40 min (c), respectively. (b) Corresponding photoluminescence (PL) images and local emission spectra of the crossbars shown in (a), demonstrating a progressive green-to-red spatial gradient as iodine ions diffuse outward from the intersection point over time. Systematic multi-temperature PL matrix tracking the spatial evolution of compositionally graded emission profiles across variable temporal intervals from 0.5 h to 2 h.

## 11. Gradient emission by solid–gas phase reaction through halide exchange

Concentration gradient of halide ion in hybrid perovskite is reported by exchange reaction in solid/gas phase by Y. Zhang, *et al.*^62^ The vapor-phase anion-exchange experiment was performed using single-crystalline CsPbBr_3_ nanoplates with different thicknesses (approximately 352–374 nm). First, the pristine nanoplates were characterized by SEM and wide-field photoluminescence (PL) imaging, and their initial green PL spectrum was recorded. The nanoplates were then exposed to sublimed *n*-C_4_H_9_NH_3_I vapor at 175–180 °C, enabling iodide ions to diffuse into the CsPbBr_3_ lattice and progressively replace bromide ions. The anion-exchange process was monitored at 10, 15, 20, and 25 min intervals using confocal PL mapping to visualize the emergence and spatial evolution of multi-color emission. At each interval, micro-PL spectra were collected from three representative locations—the face (1), edge (2), and corner (3)—to resolve position-dependent compositional changes. The corresponding lateral PL profiles were quantitatively analyzed to determine the local iodine-substitution ratio and reveal the spatial dynamics and thickness-dependent progression of vapor-phase halide exchange. Fig. 16 provides a comprehensive analysis of the anion exchange process within individual perovskite by gradient emission by solid–gas phase reaction through halide exchange.

**Fig. 16:**
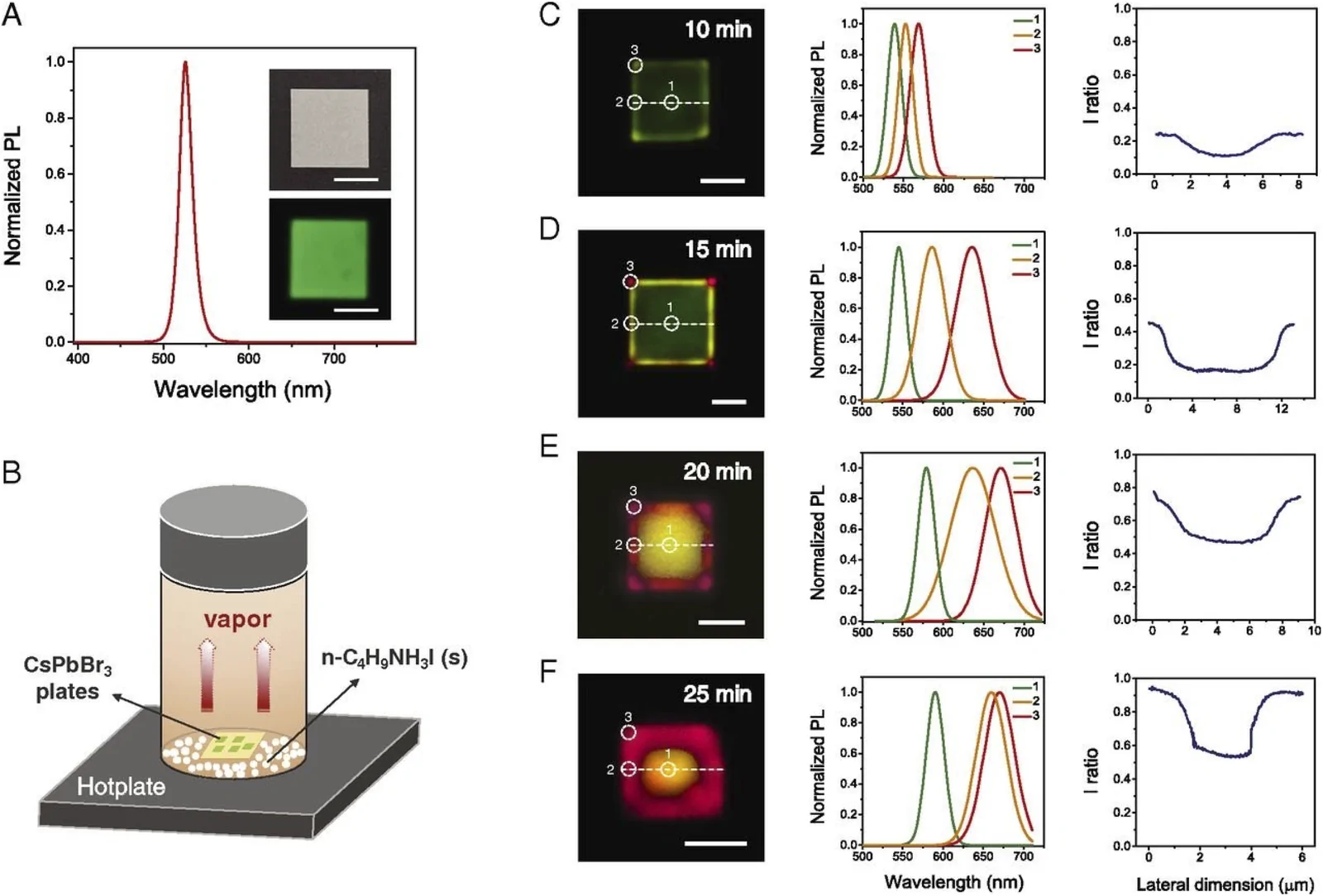
Vapor-phase anion exchange dynamics in single-crystalline CsPbBr_3_ nanoplates. (A) PL spectrum of pristine CsPbBr_3_; top inset shows SEM morphology and bottom inset shows green wide-field PL image (scale bars: 5 µm). (B) Schematic of vapor-phase conversion *via n*-C_4_H_9_NH_3_I sublimation at 175–180 °C. (C–F) Anion exchange evolutions at 10, 15, 20, and 25 min intervals for nanoplates of varying thicknesses are in 352, 374, 352, and 360 nm. Columns from left to right display: real-space confocal PL maps tracking localized multi-color emission, micro-PL emission spectra taken from the face (1), edge (2), and corner (3), and quantified lateral spatial profiles of the converted iodine substitution ratio.^62^ Reproduced from ref. 62 with permission from [PNAS] [*Proc. Natl. Acad. Sci. U. S. A.*], copyright 2026.

## 12. Vapor-based spatial halide exchange in perovskite

Vapor phase solid state perovskite preparation and synthesis were reported by Tang *et al.*^63^ The patterned PbI_2_ thin-film lines were first prepared on a SiO_2_/Si substrate and placed in a tube furnace under a controlled Ar flow. The samples were initially exposed to methylammonium iodide (MAI) vapor, enabling vapor-phase intercalation and conversion of PbI_2_ into patterned MAPbI_3_. Without removing the sample, methylammonium bromide (MABr) vapor was subsequently introduced to induce continuous *in situ* halide exchange, progressively replacing I^−^ with Br^−^ and ultimately forming MAPbBr_3_ line patterns. The morphological evolution of the patterned features was monitored by AFM at selected MABr exposure times (10, 20, and 30 min), using 100 × 100 µm^2^ topographical maps and corresponding cross-sectional height profiles measured at the sample center. These measurements enabled tracking of changes in line height, surface morphology, and structural integrity during the PbI_2_ → MAPbI_3_ → MAPbBr_3_ transformation (Fig. 17).^64^

**Fig. 17:**
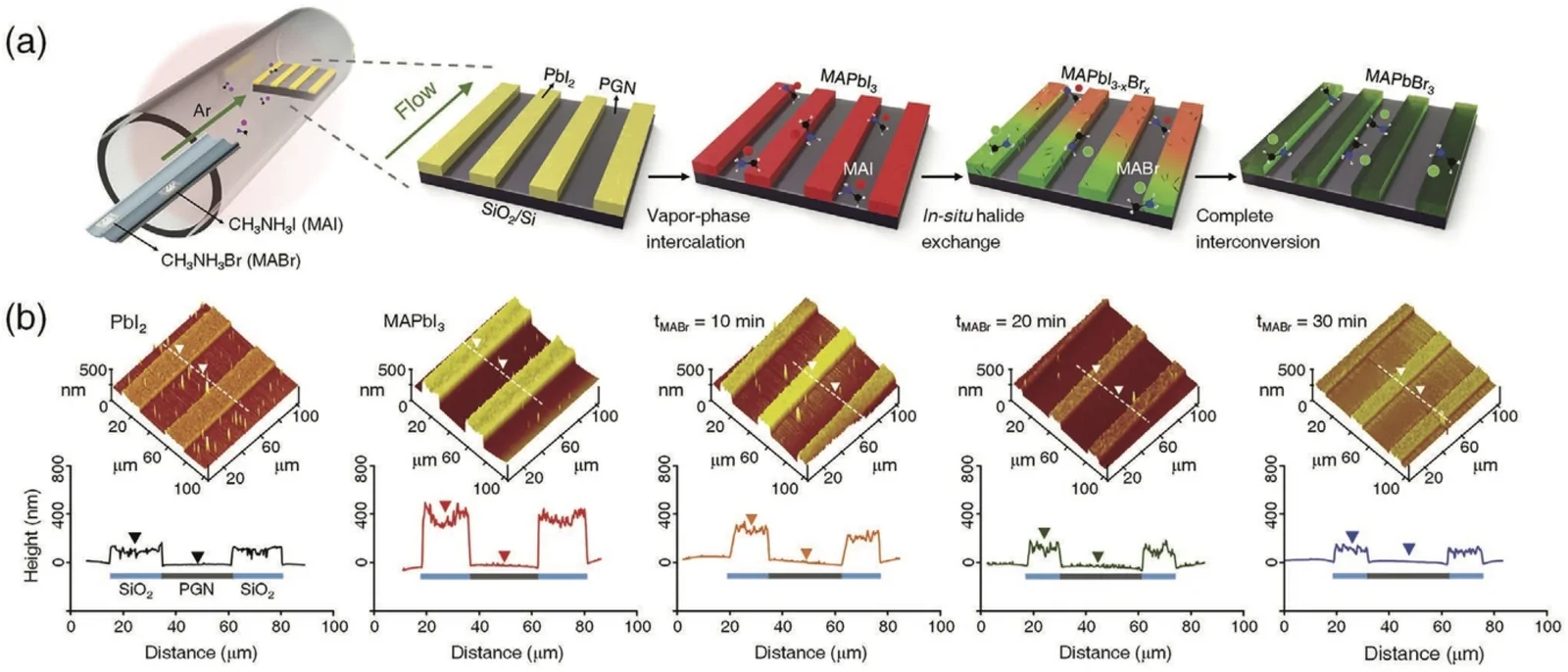
Vapor-phase conversion and *in situ* halide exchange of patterned perovskite lines. (a) Schematic illustrating the process flow inside a tube furnace under Ar flow, transitioning from patterned PbI_2_ on SiO_2_/Si to MAPbI_3_*via* vapor-phase intercalation (MAI), followed by continuous *in situ* halide exchange (MABr) to form MAPbBr_3_. (b) 3D topographical AFM mappings (100 µm × 100 µm scale) and corresponding cross-sectional height profiles tracking the evolution of line features at pristine PbI_2_, converted MAPbI_3_, and intermediate MABr exposure intervals (10, 20, and 30 min) measured at the sample center.^63^ Reproduced from ref. 63 with permission from [Wiley] [*Small*], copyright 2026.

## 13. Experiment of practical application – tailored light emission for displays and Micro-LEDs

Imagine a display where each pixel is a meticulously crafted perovskite microcrystal, emitting a specific color based on its localized halide composition.^53^ Spatially controlled halide exchange makes this vision a reality. By selectively exchanging halides within a perovskite film, researchers can create pixelated structures with varying emission colors.

For instance, studies showcase the creation of high-resolution, full-color perovskite micro-LED arrays using a photolithography-assisted halide exchange process. This approach enables precise control over the spatial distribution of chlorine, bromine, and iodine within the perovskite film, leading to the generation of red, green, and blue pixels with exceptional color purity and brightness. This paves the way for the development of next-generation displays with superior resolution, color gamut, and energy efficiency compared to conventional technologies.

From experiment to application, to showcase halide exchange process, researcher developed inkjet-printed university logo with green-emitting CsPbBr_3_ nanocrystals and selectively converted parts to red and blue using a light-source-integrated Dimatix printer and *tert*-butyl chloride (for blue) and *tert*-butyl iodide (for red) inks. The resulting trichromatic logo, achieved *via* this color-patterning method, is shown in Fig. 18. Fig. 18b demonstrates a minimum feature size of approximately 50 µm, suitable for most display applications. Importantly, the haloalkane inks vaporize completely after the exchange, leaving no residues, a significant advantage over salt-based methods that can cause haze or reverse-exchange. This convenient post-deposition color conversion approach holds promise for next-generation micro-LED and electroluminescent quantum dot displays requiring micropatterned trichromatic emission. The nanowire heterojunctions exhibit good stability. After one week of storage in a nitrogen atmosphere, confocal PL mapping of both partially bromide/chloride and superlattice nanowires showed PL emissions and sharp junctions comparable to their initial states. These results indicate significantly slower anion interdiffusion across the heterojunctions compared to the exchange reaction at the solid–liquid interface.

**Fig. 18:**
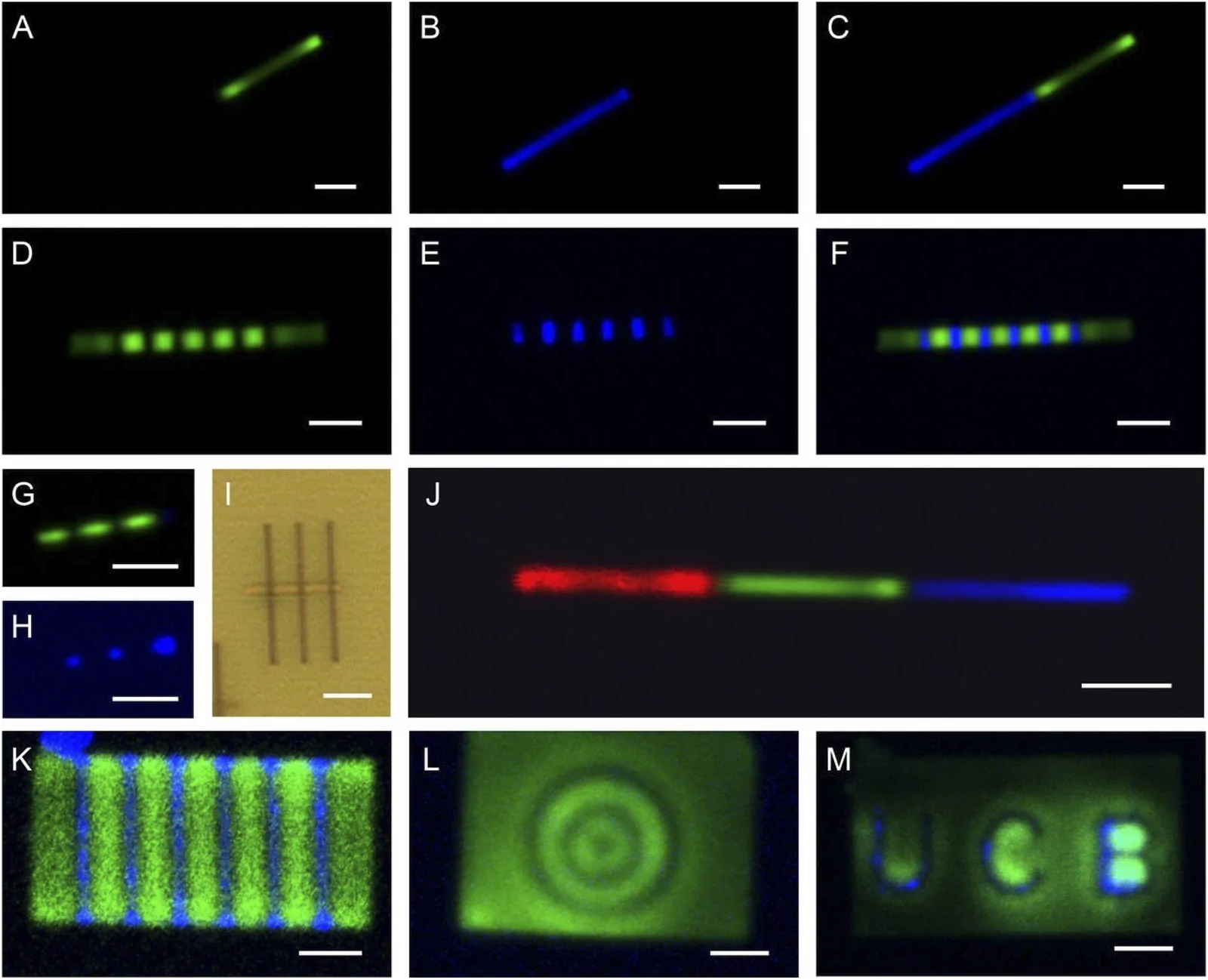
Confocal photoluminescence (PL) mapping of various heterojunction nanowires and patterns. (A–C) PL map of a partially bromide/chloride nanowire (blue: 410–450 nm, green: 500–550 nm). (D–F) PL map of a bromide–chloride superlattice nanowire (pixel size < 1 µm). (G–I) PL map and optical image of a bromide–chloride superlattice nanowire (pixel size < 500 nm). (J) PL map of a three-color heterojunction nanowire (blue: 410–450 nm, green: 500–550 nm, red: 580–640 nm). (K–M) PL maps of different patterns on CsPbBr_3_ plates (scale bars: 3 µm).^51^ Reproduced from ref. 51 with permission from [PNAS] [*Proc. Natl. Acad. Sci. U. S. A.*], copyright 2026.

## 14. Experiment of practical application: micro-lasers with defined modes and emission patterns by halide exchange reaction

Perovskites hold immense promise for the development of miniaturized lasers with exceptional properties. Spatially controlled halide exchange offers a powerful tool for engineering the emission characteristics of these micro-lasers. By manipulating the halide composition within specific regions of the perovskite microcavity, researchers can achieve precise control over the lasing modes and emission patterns.^63,65^ A recent study demonstrated the fabrication of micro-lasers with spatially defined lasing modes using a laser-assisted halide exchange technique.^65^ This approach enabled the creation of micro-lasers with single-mode emission, a crucial property for high-performance lasers. Additionally, spatially patterned halide exchange allows for the creation of micro-lasers with tailored emission patterns, such as doughnut-shaped modes or directional emission profiles, opening doors for diverse applications in optical communication and sensing.

Fig. 19–21 demonstrated the experimental work which shows that compositionally graded perovskite nanowires can exhibit spatially varying emission and lasing behavior.^63,65^ These structures establish a direct link between halide-exchange-induced bandgap gradients and optical functionality. The experiment demonstrates a facile vapor-phase route for producing composition-graded cesium lead halide perovskite (CsPbBr_*x*_I_3−*x*_) nanowires (NWs) with spatially tunable optical emission and dual-color lasing. In the synthesis, CsI powder was placed at the center of a home-built chemical-vapor-deposition system, while PbBr_2_ and PbI_2_ (1 : 1 molar ratio) were positioned upstream; freshly cleaved muscovite mica served as the downstream substrate, with Ar flowing at 40 torr. At 450 °C, 20 min deposition produced highly oriented, triangular-prismatic NWs extending to hundreds of micrometers with widths of 300 nm–1.5 µm. EDS revealed a pronounced axial compositional gradient, with the Br/I ratio increasing from approximately 1.5 : 1.5 at the center to 2.9 : 0.1 at the ends, while HRTEM, SAED, and XRD confirmed the cubic perovskite structure and [001]-oriented growth. Time-dependent experiments revealed the origin of this gradient: I-rich CsPbBr_*x*_I_3−*x*_ initially deposits because PbI_2_ has higher vapor pressure/lower evaporation temperature than PbBr_2_, followed by progressive Br incorporation and anion exchange as growth proceeds. Temperature was therefore critical; insufficient temperature produced little material, whereas 530 °C favored homogeneous rather than graded NWs. This spatial compositional modulation generated a corresponding bandgap gradient, producing a strong position-dependent PL blueshift from ∼550 nm at the I-rich center to ∼510 nm at Br-rich ends. Under 400 nm, 150 fs pulsed excitation, distinct lasing occurred at 521 nm at the Br-rich end and 556 nm at the center, while an intermediate region simultaneously generated both wavelengths. The dual-color lasing exhibited a 35 nm separation, with thresholds of ∼16 and 28 µJ cm^−2^, demonstrating that a single composition-graded NW can function as a spatially addressable, multicolor nanoscale laser. By contrast, fully integrated on-chip multi-wavelength lasers, quantum-photonic circuits, and complex micro-LED architectures based specifically on spatial halide exchange remain prospective device directions rather than established demonstrations in the literature reviewed here.

**Fig. 19:**
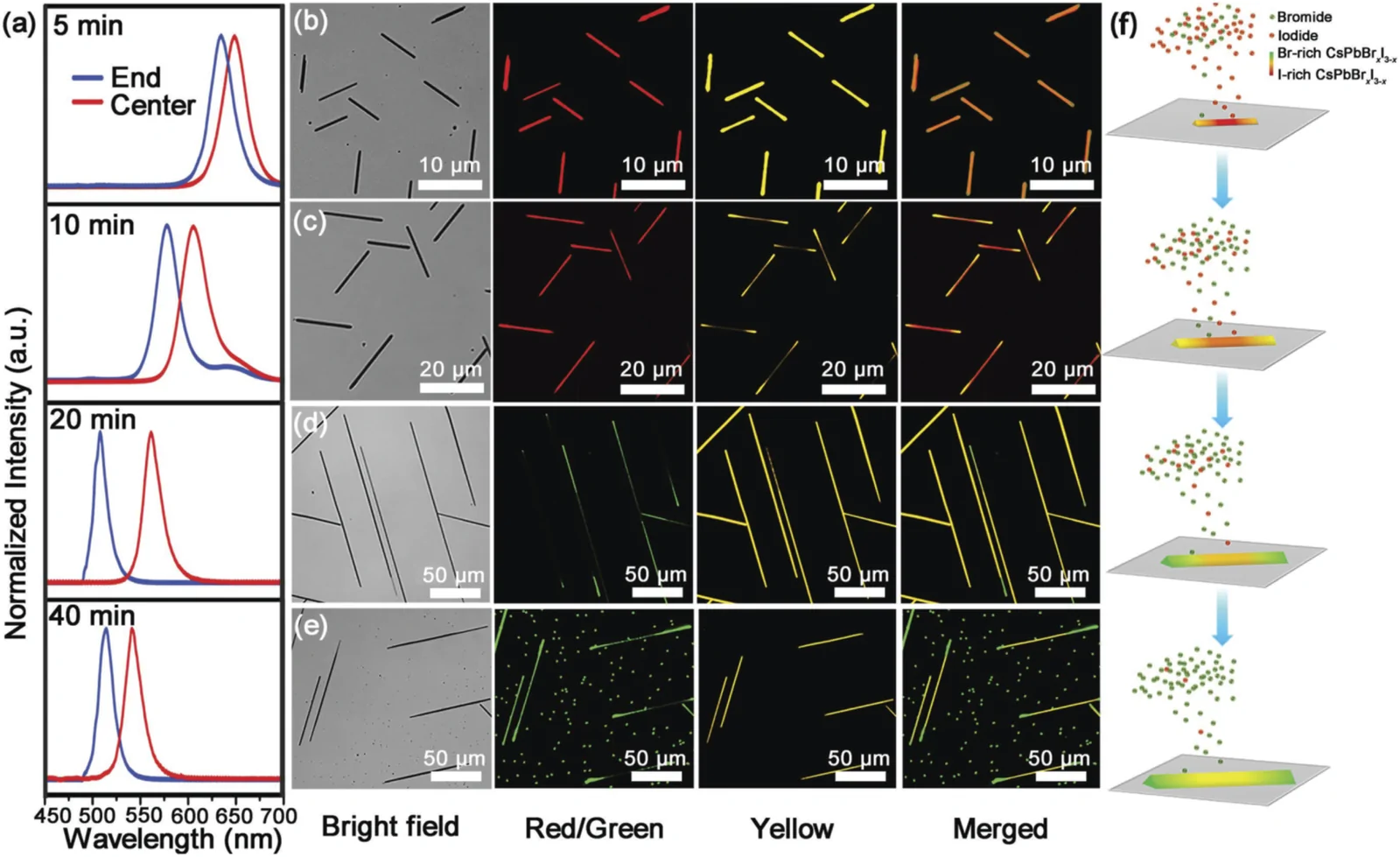
Composition-graded cesium lead halide (CsPbBr_{3−*x*}_I_*x*_) perovskite nanowires: (a) Normalized emission spectra from the end and center of nanowires at different halide-exchange times (5 min, 10 min, 20 min, and 40 min); (b–e) corresponding bright-field, fluorescence (red/green, yellow), and merged optical microscopy images at 5 min, 10 min, 20 min, and 40 min of reaction; and (f) schematic illustration of the halide exchange process showing the diffusion and gradual compositional variation along the nanowire.^65^ Reproduced from ref. 65 with permission from [Wiley] [*Advanced Materials*], copyright 2026.

**Fig. 20:**
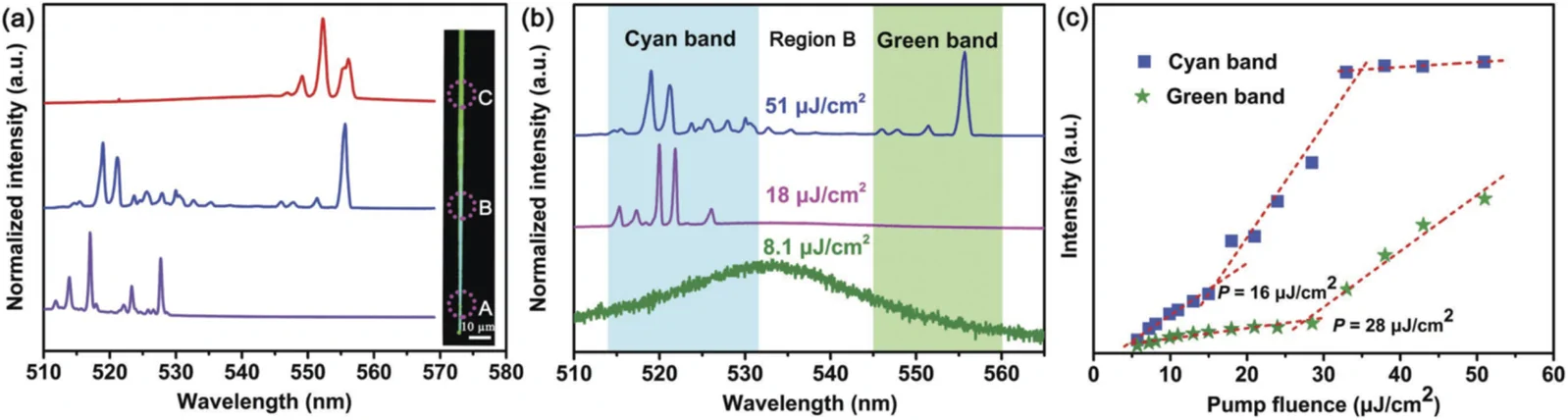
Position-dependent and dual-color lasing performance of composition-graded cesium lead halide (CsPbBr_{3−*x*}_I_*x*_) perovskite nanowires: (a) spatially resolved emission spectra collected from different regions (A, B, and C) along a single nanowire, with the inset showing the corresponding optical/fluorescence image and probed spots (10 µmm scale). (b) Normalized emission spectra under increasing excitation pump fluences (8.1 µJ cm^−2^, 18 µJ cm^−2^, and 51 µJ cm^−2^), demonstrating the evolution from spontaneous emission to dual-color lasing in the cyan and green bands. (c) Integrated emission intensity as a function of pump fluence for the cyan band (threshold *P* = 16 µJ cm^−2^) and green band (threshold *P* = 28 µJ cm^−2^).^65^ Reproduced from ref. 65 with permission from [Wiley] [*Advanced Materials*], copyright 2026.

**Fig. 21:**
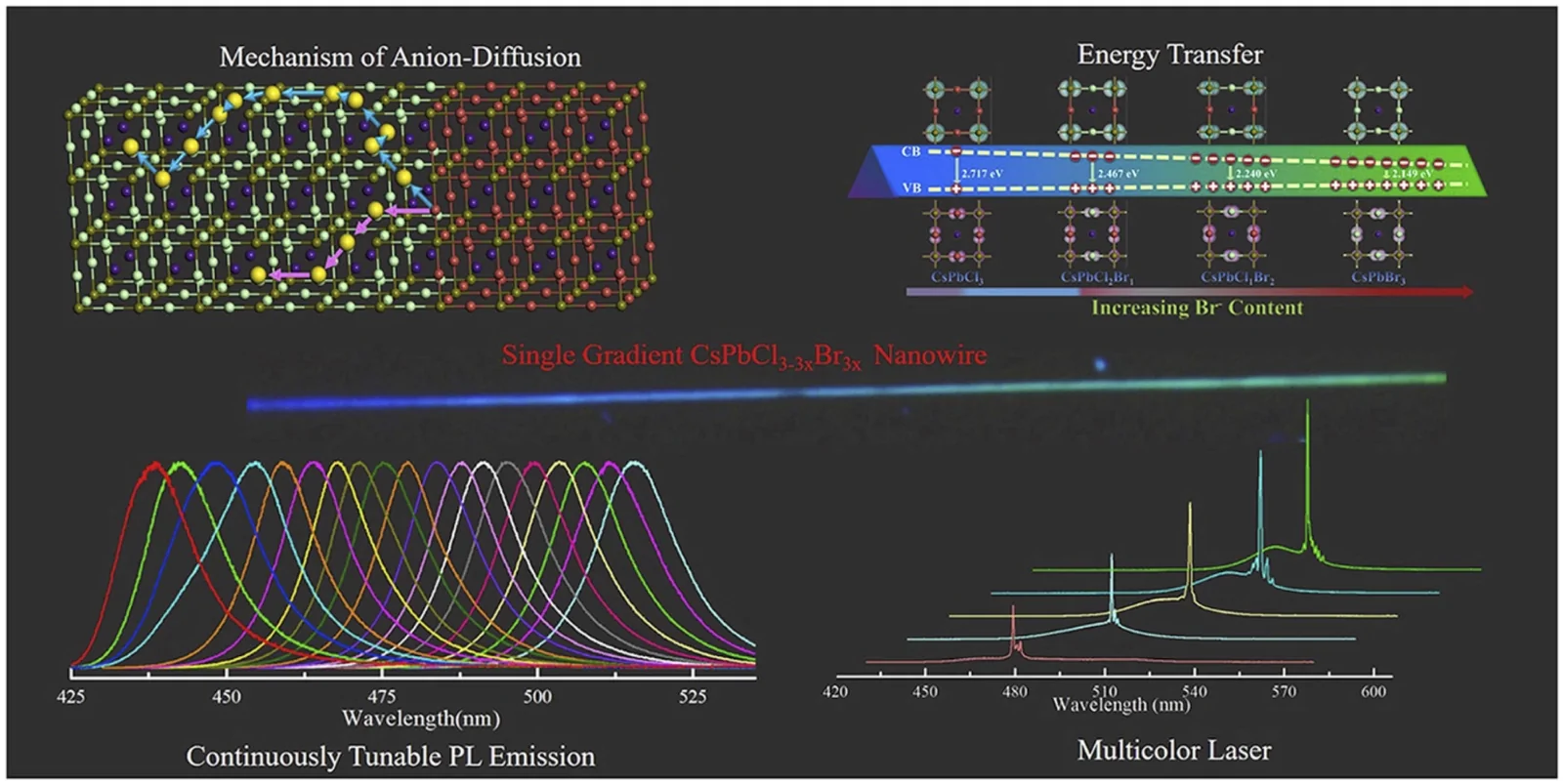
Mechanism and output of single gradient CsPbCl_{3−*x*}_Br_*x*_ perovskite nanowires.^68^ Reproduced from ref. 65 with permission from [Elsevier] [*Nano Energy*], copyright 2026.

Fig. 21 showed the mechanism, energy band engineering, and optical performance of a single gradient CsPbCl_{3−*x*}_Br_{3*x*}_ perovskite nanowire which are driven by anion-diffusion processes during halide exchange within the crystal lattice. Through energy band engineering, the conduction band (CB) and valence band (VB) alignments shift continuously as a function of increasing Br content, transitioning from CsPbCl_3_ to CsPbBr_3_. Optical and fluorescence microscopy reveal the distinct spatial features of an individual composition-graded nanowire, which supports continuously tunable photoluminescence (PL) emission wavelengths spanning from ∼430 nm to ∼525 nm. Additionally, this gradient architecture enables multicolor lasing output, operating at distinct discrete wavelengths of ∼480 nm, ∼510 nm, ∼540 nm, and ∼570 nm.

## 15. Experiment of practical application: mixed halide perovskite solar cell by halide exchange reaction

Halide gradient concentration in hybrid perovskite effect can be found in the solar cell efficiency. Fig. 22 and 23 show the mechanism of halide concentration grading in hybrid perovskites which is established through a partial ion-exchange approach, where an organic bromide (such as MABr or FABr) is spin-coated onto a primary absorber like MAPbI_3_ or FAPbI_3_ and subsequently thermally annealed to induce a continuous decrease in bromine (Br) concentration from the front to the rear interface.^66^ Because increasing Br content downshifts the valence band maximum while leaving the conduction band minimum relatively unchanged, this compositional variation creates a bandgap grading that introduces an additional internal electric force. This electric field assists the drift of photogenerated holes away from the front contact (the perovskite/PCBM interface), thereby lowering the local concentration of holes directly at that interface. As a result, interface recombination is significantly suppressed—lowering the reverse saturation current density—which concurrently boosts the open-circuit voltage (*V*_OC_), short-circuit current density (*J*_SC_), and overall power conversion efficiency of the device.^67^

**Fig. 22:**
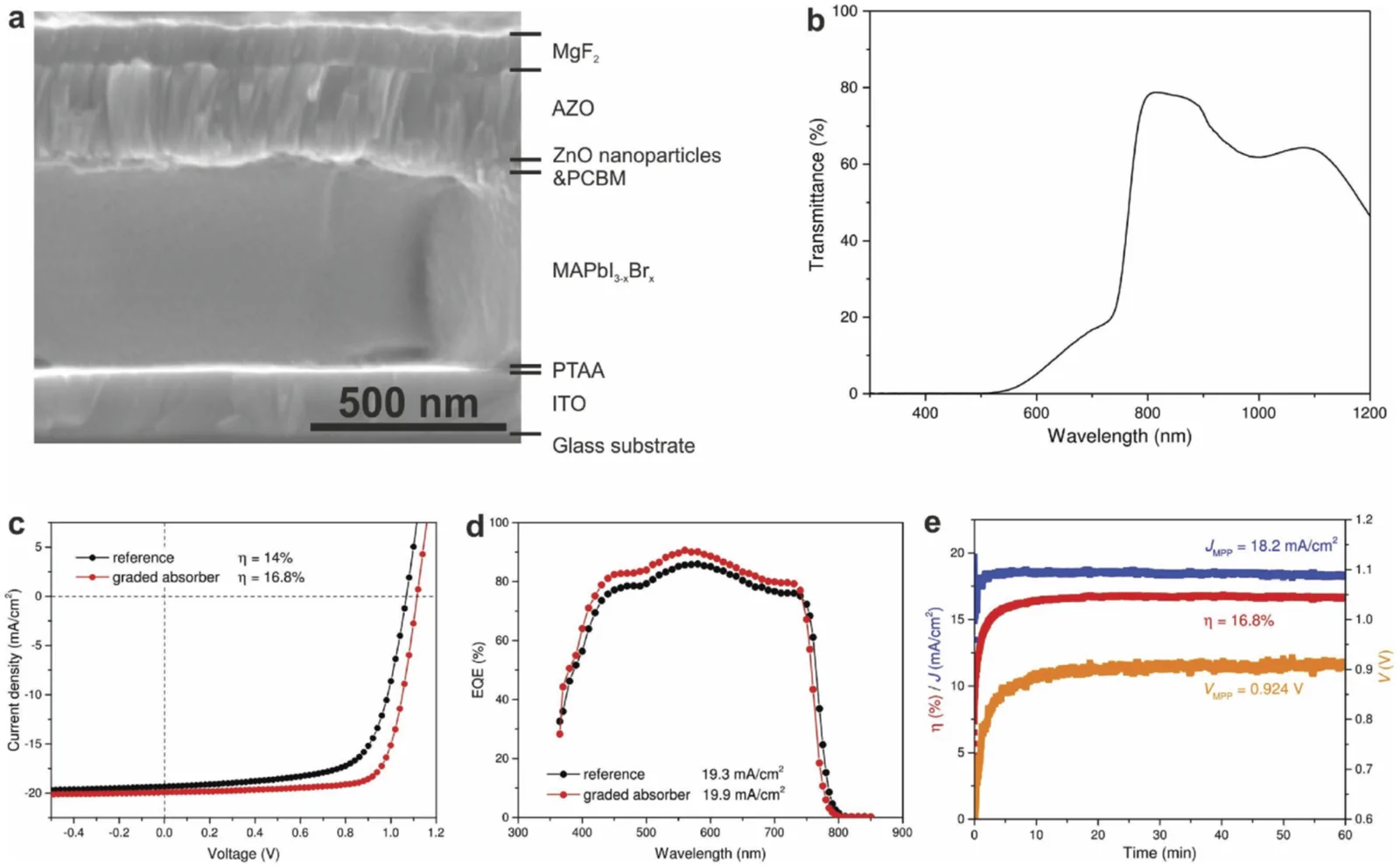
Cross-sectional structure, optical properties, and photovoltaic performance of halide-graded MAPbI_{3−*x*}_Br_*x*_ perovskite solar cells. (a) SEM image, (b) trarnsmittance spectrum, (c) current intensity, (d) external quantum efficiency and (e) output performance.^66^ Reproduced from ref. 66 with permission from [Wiley] [*Advanced Science*], copyright 2026.

**Fig. 23:**
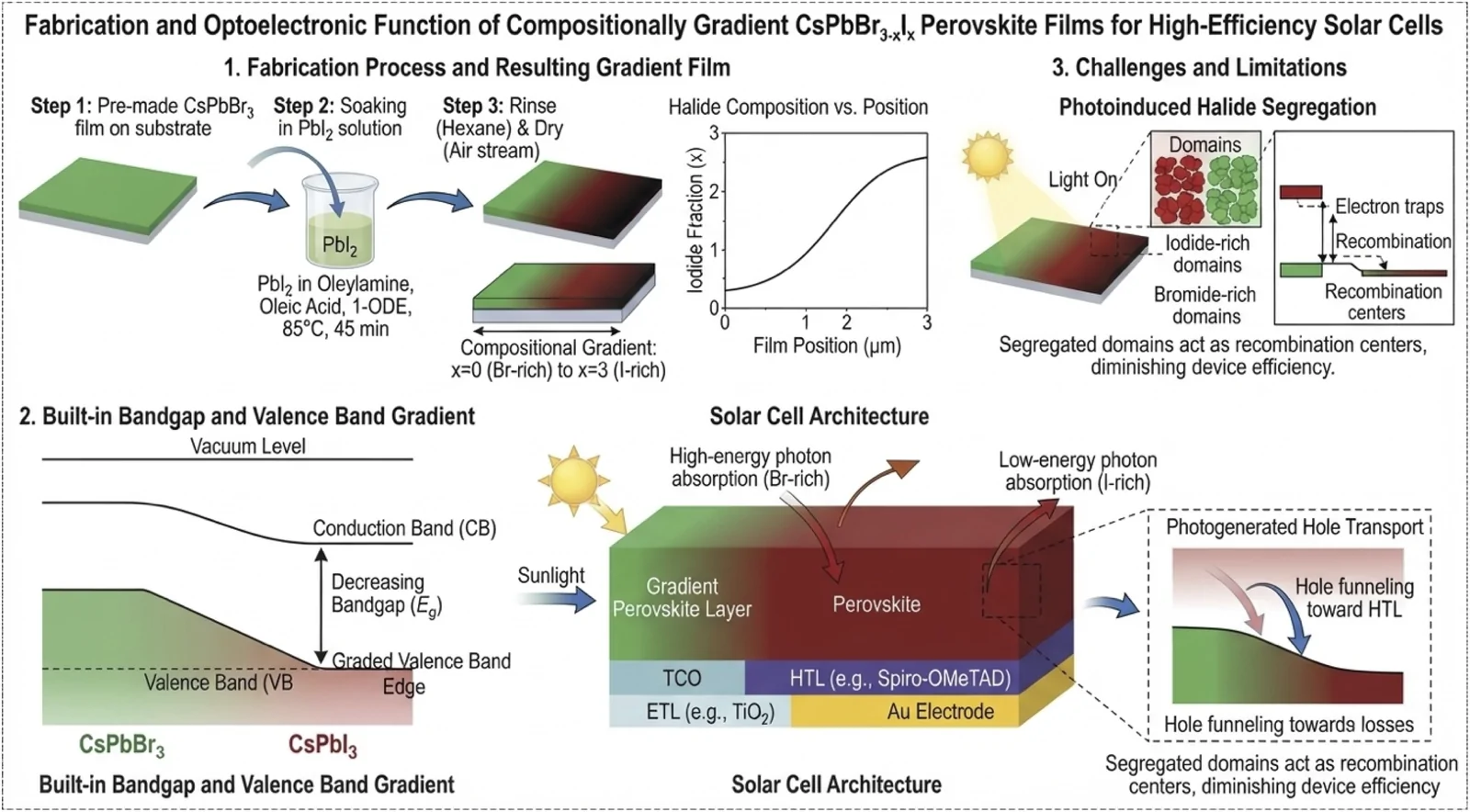
Structural and optical evolution of gradient halide perovskite films, schematic illustration of a compositionally graded perovskite architecture showing the transition from a bromide-rich region (CsPbBr_3_) to an iodide-rich region, with respective material phases represented for CsPbI_3_ (red), CsPbBr_3−*x*_I_*x*_ (orange), under illumination.

In the report by Scheidt and Kamat (2019),^68^ gradient halide perovskite (CsPbBr_3−*x*_I_*x*_) films are achieved by soaking halide exchange mechanism by pre-made CsPbBr_3_ films in a lead(ii) iodide (PbI_2_) solution (prepared in oleylamine, oleic acid, and 1-octadecene) cooled to 85 °C for a reaction time of 45 minutes, after which the film is rinsed thoroughly with hexane and dried with an air stream. This process creates a systematic variation in halide composition where iodide concentration ranges from *x* = 0 to *x* = 3. This built-in compositional and bandgap gradient affects solar cell functionality by enabling the integrated capture of visible photons across the spectrum while minimizing photon losses. Higher-energy photons are effectively absorbed in the CsPbBr_3_-rich region, while lower-energy photons pass through to be absorbed in the energetically low-lying, iodide-rich regions. Additionally, the shift in the valence band (resulting from the high contribution of halide p-orbitals) helps preferentially shuttle photogenerated holes toward the CsPbI_3_-rich regime and out toward the hole transport layer, minimizing recombination losses. Conversely, the mobility of halide ions in mixed halide architectures can lead to photoinduced segregation into iodide- and bromide-rich domains that act as recombination centers, which can diminish overall device efficiency.

Spatially controlled halide exchange can directly create compositionally graded heterojunctions within a single perovskite crystal or nanowire. This functionality has been experimentally demonstrated through localized anion exchange and source-limited solid-state diffusion, producing spatially resolved PL profiles and composition gradients. Such structures provide a credible materials platform for built-in bandgap gradients, carrier funneling, and wavelength-selective optical functions. Device concepts such as patterned charge-transport pathways and integrated photonic circuits are retained only as proposed applications unless spatial halide exchange has been directly demonstrated.

Recently spatially controlled halide exchange highlight major breakthroughs and ongoing challenges in the engineering of hybrid perovskites for optoelectronic applications. Methods enabling spatial halide exchange—such as remote or optically guided exchange—allow for the fabrication of tailored heterojunctions and precise bandgap modulation within a single perovskite crystal or film. For instance, site-specific halide exchange successfully creates perovskite heterojunctions with spatially modulated optical properties, though it faces challenges in delivering halide ions with high selectivity to targeted domains.^47–49^

Advanced strategies enable reversible, full-range bandgap tuning through exchange reactions, as demonstrated in MAPbBr_3−*x*_Cl_*x*_ and MAPbBr_3−*x*_I_*x*_ nanocrystals, far surpassing traditional compositional tuning.^51^

Key to the materials' optoelectronic performance, the spatial arrangement of octahedral units and the use of thermally activated exchange have both been shown to dynamically and reversibly tailor band structure and photoluminescence. These approaches exploit the inherent lattice flexibility and low activation energies of halide ions in the perovskite matrix, capitalizing on vacancy-assisted diffusion and interfacial phenomena to achieve localization and gradient formation.^65,68^

## 16. Challenges and future perspectives: advancing halide exchange in perovskites

Despite the immense potential of halide exchange reactions in perovskites, several challenges remain to be addressed for their successful integration into next-generation optoelectronic devices.

### 16.1 Achieving high spatial resolution for precise control

A critical challenge lies in achieving high spatial resolution during halide exchange. Conventional methods, like solution-based immersion, often lead to uniform changes throughout the perovskite film, limiting the ability to create intricate patterns or localized modifications. Recent advancements in laser trapping offer a promising solution. Studies by demonstrate the use of tightly focused laser beams to manipulate halide precursors within a solution, enabling localized exchange reactions on a single crystal level.^49,51^ This technique has the potential to create perovskite structures with spatially varying emission profiles, ideal for micro-LED displays or patterned optoelectronic devices. Further research is needed to refine laser trapping techniques and explore other methods, such as focused ion beam irradiation or microfluidic channels, to achieve even finer control over the spatial resolution of halide exchange. Fig. 24 shows the proposed scheme of spatial halide exchange for future LED light.

**Fig. 24:**
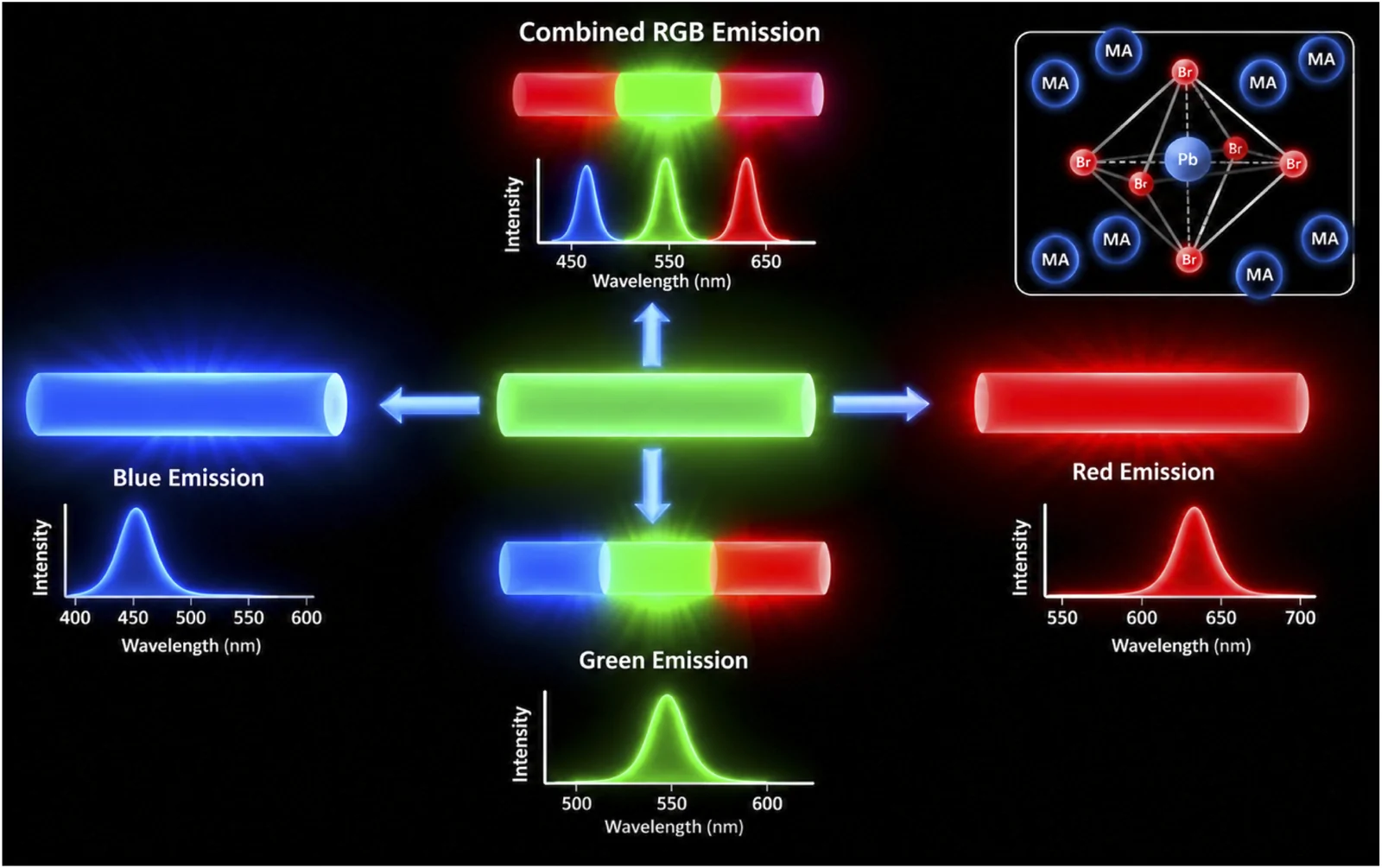
Proposed scheme of spatial halide exchange for future LED light.

### 16.2 Pattern stability and long-term compositional fidelity

The stability of a spatially exchanged region has two distinct dimensions: chemical stability of the halide distribution and optical stability of the associated emission. The reviewed literature provides encouraging short-term evidence but does not yet establish a common long-term stability protocol. A representative nanowire study reported that spatially resolved PL features remained observable after one week of storage in nitrogen, supporting the persistence of the patterned composition under inert conditions. By contrast, many laser-, solution-, and vapor-mediated studies characterize the pattern immediately after exchange or over the reaction period without reporting extended aging, humidity exposure, thermal cycling, or repeated optical excitation. These gaps make direct comparison of pattern lifetime difficult.^65,68,69^

Mixed-halide perovskites are intrinsically susceptible to ion migration and light- or bias-induced compositional redistribution. Consequently, a pattern that is initially spatially resolved may broaden or evolve during operation if the local chemical potential, defect population, temperature, or illumination changes. Stabilization strategies should therefore be evaluated specifically at the patterned interface, rather than only on the unexchanged material. Future reports should quantify PL-map retention, composition-map retention (EDS/XPS or equivalent), and feature broadening after defined thermal, humidity, illumination, and electrical stress tests. Recent work on ion-migration suppression and lead-free/pseudohalide perovskites provides useful broader design principles, but these approaches should not be assumed to stabilize a spatial halide-exchange pattern unless tested directly.^70–72^

### 16.3 Scalability for device fabrication

While halide exchange has been successfully demonstrated on a small scale, scaling up the process for large-scale device fabrication presents another challenge.^73–75^ Traditional solution-based methods often face issues with uniformity and control over exchange across larger areas. Microfluidic platforms offer a promising avenue for scalable halide exchange.^76^ By precisely controlling the flow of precursor solutions and manipulating reaction conditions within microfluidic channels, researchers can achieve more uniform and controlled exchange processes across larger device areas. Additionally, integrating laser trapping techniques with microfluidic platforms could potentially enable a unique combination of high spatial resolution and scalability for large-scale device fabrication.

At the end, Table 2 provides a comparative overview of spatially controlled halide-exchange strategies in metal-halide perovskites, emphasizing the relationship between perovskite morphology, exchange chemistry, localization strategy, spatial resolution, processing time, photoluminescence (PL) response, stability, and the principal limitations of each approach. The halide exchange has evolved from relatively uncontrolled bulk compositional modification toward increasingly localized and programmable approaches capable of generating microscale, nanoscale, and gradient optical features. For CsPbBr_3_ nanocrystals, liquid–solid/light-mediated exchange using haloalkanes and Br → Cl/I substitution produces visible-range multicolor PL, while photocatalytic or light-patterning approaches provide nanocrystal-scale localization; however, the table identifies surface chemistry and spatial confinement as major limitations because the exact feature width was not reported.^30^ This behavior is consistent with the established sensitivity of lead–halide perovskites to anion migration and exchange, in which the substitution of Br^−^ by the smaller Cl^−^ or larger I^−^ ion modifies lattice dimensions and electronic band structure, thereby shifting the PL wavelength. Recent literature further confirms that halide exchange in CsPbBr_3_ is governed by vacancy-assisted ion migration and can produce rapid, composition-dependent optical shifts. A particularly important advance is represented by MAPbBr_3_ single crystals (20 × 20 µm^2^), where a 1064 nm near-infrared laser was employed to optically trap the halide precursor and confine the exchange to a localized micrometer-scale region.^53^ The Table 2 reports approximately 1 min for bulk exchange and 30 min for localized irradiation, accompanied by a PL transformation from approximately 549 nm (green) toward the red region, corresponding to formation of MAPb(Br_*x*_I_1−*x*_)_3_. This result is particularly significant because optical trapping increases the local concentration of the reactive halide precursor while suppressing undesired exchange elsewhere; the original report explicitly demonstrated site-selective bandgap and emission-color tuning using a 1064 nm continuous-wave laser, with reduced photothermal and photochemical damage. Similar laser-assisted processing of MAPbBr_3_ microrods produced localized green-to-red PL evolution, with the table reporting approximately 540 nm to 750 nm and processing on the scale of tens of minutes. The broader significance of this red shift is that incorporation of I^−^ progressively narrows the bandgap relative to Br-rich MAPbBr_3_, enabling spatially programmable emission rather than merely uniform compositional conversion. Importantly, the table also highlights a strategy based on laser-assisted vacancy filling, in which sequential MABr/MAI treatment creates a locally inhibited exchange region; approximately 20 min of MABr treatment preserves the green-emitting region and suppresses subsequent exchange, demonstrating that vacancy engineering can be used as a chemical “mask” for spatially selective ion migration.^48,49^

The Table 2 further demonstrates that physical masking and lithographic patterning provide an alternative route to high-fidelity spatial composition control. In CsPbBr_3_ nanowires, oleylammonium chloride/iodide exchange through an electron-beam-lithography-defined PMMA opening generates micrometer-scale Br/Cl or Br/I compositional gradients, with green-to-blue or green-to-NIR PL responses, respectively; representative nanowires retained their spatial PL patterns for one week under N_2_, although interdiffusion broadened the interfaces and EBL remained a relatively low-throughput technique. Such behavior agrees with broader demonstrations that halide exchange can be used to construct spatially varying optical properties in one-dimensional perovskite structures and that the resulting compositional gradients directly translate into spatial PL gradients.^77^ For CsPbBr_3_ thin films, controlled dipping or withdrawal with CsI/CsCl produces continuous multicolor emission gradients across the film; importantly, the exchange time and spatial resolution were not quantitatively specified, indicating that diffusion remains a major factor governing boundary sharpness.^64^ A related solid–solid strategy based on direct interfacial contact between CsPbCl_3_ and CsPbBr_3_ nanowires provides tunable gradient lengths and yields deep-blue-to-green spatial PL gradients that remain stable for several days at room temperature, although relatively slow kinetics and spontaneous diffusion limit rapid pattern formation. Particularly notable is the CsPbBr_3_/δ-CsPbI_3_ nanowire crossbar, where a source-limited iodine supply creates a spatially constrained exchange geometry over tens of micrometers, with a reported processing window of 20–90 min at 200–240 °C and a pronounced green → yellow → red → NIR emission progression.^78,79^ These data demonstrate that both chemical composition and optical output can be continuously programmed along a single micro/nanostructure, although elevated temperature and diffusion control are necessary.

Vapor-phase approaches provide another important dimension of spatial control. The Table 2 reports that CsPbBr_3_ nanoplates exposed locally to *n*-C_4_H_9_NH_3_I vapor exhibit thickness-dependent exchange regions spanning approximately 20–380 nm, with exchange occurring within 10–25 min and producing longer-wavelength iodine-rich PL relative to the original green emission. The strong dependence on thickness, edge geometry, and vapor exposure emphasizes that halide diffusion is controlled not only by intrinsic ion mobility but also by the local geometry and surface-accessibility of the perovskite. Finally, patterned MAPbI_3_/MAPbBr_3_ thin films generated through sequential MAI and MABr vapor-phase exchange demonstrate µm-scale patterned lines and I-rich-to-Br-rich spectral evolution within approximately 10–30 min of MABr exposure; however, pattern fidelity remains constrained by vapor diffusion. Overall, the analytical data summarized in the Table 2 establish a clear processing–performance hierarchy: laser/optical trapping offers highly localized and dynamically addressable exchange; lithographic masking provides relatively high pattern fidelity but lower throughput; liquid-phase dipping enables continuous large-area gradients but suffers from diffusion; solid–solid interfaces offer chemically stable gradients but slower kinetics; and vapor-phase exchange permits nanoscale-to-microscale modification but remains sensitive to thickness and geometry. The resulting PL shifts—from approximately 549 nm green emission to ∼750 nm red emission, and further into the NIR for I-rich compositions, or toward blue emission for Cl-rich compositions—provide a convenient optical readout of local halide composition. This is consistent with experimental demonstrations that CsPbBr_3_ films subjected to CsI or CsCl dipping develop continuous green-to-red or green-to-blue PL gradients, respectively, because the local halide composition determines the bandgap. Thus, the Table 2 collectively indicates that the central challenge is no longer simply achieving halide exchange, but controlling ion-migration length scales, exchange kinetics, interface sharpness, vacancy concentration, and long-term compositional stability simultaneously.

## 17. Conclusion and future outlook

Spatial halide exchange provides a distinctive route to sculpting optical properties within individual perovskite crystals, nanowires, nanoplates, and patterned films. The central lesson from the literature is that spatial functionality is governed by the competition between localization and ion transport: optical trapping or localized photochemistry can confine the reaction source, lithographic masks can define the access geometry, while vacancy-assisted diffusion and interfacial transport ultimately determine how far the exchanged composition propagates. These mechanisms explain why high spatial precision is often accompanied by limited throughput, whereas diffusion- and vapor-based approaches can produce larger-area gradients but with less abrupt boundaries.

The comparative analysis also shows that the evidence base is uneven. Experimentally demonstrated achievements include localized multicolor photoluminescence, compositionally graded nanowire heterojunctions, laser-assisted suppression of exchange, and vapor- or solution-mediated spatial gradients. In contrast, several frequently cited device opportunities—fully integrated micro-LED arrays, complex photonic circuits, quantum-photonic architectures, and some solar-cell concepts—remain proposed or are based on related perovskite patterning rather than directly demonstrated spatial halide exchange. Distinguishing these evidence levels is essential for a realistic assessment of the field.

Future progress should prioritize four measurable targets: (1) sub-micrometre chemical localization with independently verified composition maps; (2) standardized reporting of exchange time, feature size, and spectral shift; (3) quantitative stability testing of patterned regions under light, heat, humidity, and electrical bias; and (4) scalable delivery platforms, including microfluidics and parallel optical/lithographic processing, that preserve compositional fidelity. A unified benchmarking framework such as Table 2 can help separate genuine advances in spatial halide exchange from improvements that arise primarily from conventional bulk exchange or generic perovskite patterning. These developments would move the field from proof-of-concept spatial color manipulation toward reproducible, device-relevant control of light.

## Author contributions

Md. Jahidul Islam: conceived the study, performed the literature review and analysis, developed the conceptual framework, interpreted the scientific findings, prepared the figures and illustrations, supervised the study, wrote the original manuscript draft, revised the manuscript, and approved the final version for publication. Ajoy Kumer: contributed to the critical interpretation of the literature, reviewed and edited the manuscript. All authors discussed the results, contributed to the intellectual content of the manuscript, read and approved the final manuscript, and agreed to be accountable for all aspects of the work.

## Conflicts of interest

Authors declare no competing interest for this research.

## Data Availability

No primary research results, software or code have been included and no new data were generated or analysed as part of this review.
